# Fast satellite DNA evolution in *Nothobranchius* annual killifishes

**DOI:** 10.1007/s10577-023-09742-8

**Published:** 2023-11-21

**Authors:** Anna Voleníková, Karolína Lukšíková, Pablo Mora, Tomáš Pavlica, Marie Altmanová, Jana Štundlová, Šárka Pelikánová, Sergey A. Simanovsky, Marek Jankásek, Martin Reichard, Petr Nguyen, Alexandr Sember

**Affiliations:** 1 Institute of Animal Physiology and Genetics, Czech Academy of Sciences, Liběchov, Czech Republic; 2 Faculty of Science, University of South Bohemia, České Budějovice, Czech Republic; 3 Department of Genetics and Microbiology, Faculty of Science, Charles University, Prague, Czech Republic; 4 Department of Experimental Biology, Genetics Area, University of Jaén, Jaén, Spain; 5 Department of Zoology, Faculty of Science, Charles University, Prague, Czech Republic; 6 Department of Ecology, Faculty of Science, Charles University, Prague, Czech Republic; 7 Severtsov Institute of Ecology and Evolution, Russian Academy of Sciences, Moscow, Russia; 8 Institute of Vertebrate Biology, Czech Academy of Sciences, Brno, Czech Republic; 9 Department of Ecology and Vertebrate Zoology, University of Łódź, Łódź, Poland; 10 Department of Botany and Zoology, Faculty of Science, Masaryk University, Brno, Czech Republic

**Keywords:** Centromere drive, Constitutive heterochromatin, RepeatExplorer, Repetitive sequences, satDNA

## Abstract

Satellite DNA (satDNA) is a rapidly evolving class of tandem repeats, with some monomers being involved in centromere organization and function. To identify repeats associated with (peri)centromeric regions, we investigated satDNA across Southern and Coastal clades of African annual killifishes of the genus *Nothobranchius*. Molecular cytogenetic and bioinformatic analyses revealed that two previously identified satellites, designated here as NkadSat01-77 and NfurSat01-348, are associated with (peri)centromeres only in one lineage of the Southern clade. NfurSat01-348 was, however, additionally detected outside centromeres in three members of the Coastal clade. We also identified a novel satDNA, NrubSat01-48, associated with (peri)centromeres in *N. foerschi*, *N. guentheri*, and *N. rubripinnis*. Our findings revealed fast turnover of satDNA associated with (peri)centromeres and different trends in their evolution in two clades of the genus *Nothobranchius*.

**Supplementary Information:**

The online version contains supplementary material available at 10.1007/s10577-023-09742-8.

## Introduction

African killifishes from the genus *Nothobranchius* Peters, 1868 (Aplocheiloidei: Nothobranchiidae) are small freshwater fishes with bigger and more colorful males compared to smaller and dull females (Wildekamp 2004; Berois et al. 2016). The genus is monophyletic and currently comprises over 90 species (Nagy and Watters 2021; Fricke et al. 2023) partitioned into seven evolutionary lineages (van der Merwe et al. 2021). *Nothobranchius* spp. are adapted to periodic droughts in south-eastern African savannahs, where they survive in isolated pools, temporarily flooded by rainwater (Blažek et al. 2013; Cellerino et al. 2016; Furness 2016). Having the shortest life cycle among vertebrates, the turquoise killifish *N. furzeri *became a popular model system for aging research (Cellerino et al. 2016; Hu and Brunet 2018). In addition, the unique biology of killifishes offers many advantages for studies related to developmental biology, population dynamics, and evolution (Cellerino et al. 2016; Terzibasi Tozzini and Cellerino 2020). For instance, their mating system and sexual dimorphism make them attractive for studies of reproductive isolation and sexual selection (Berois et al. 2016; Cellerino et al. 2016).

*Nothobranchius* killifishes became of interest also to genome and sex chromosome research. Studies reported high repetitive DNA content in *Nothobranchius* genomes (Reichwald et al. 2009, 2015; Cui et al. 2019; Štundlová et al. 2022) and wide variation in diploid chromosome numbers (2n = 16–50) and karyotype structures in 73 studied representatives (Krysanov et al. 2016, 2023; Krysanov and Demidova 2018). Moreover, a multiple sex chromosome system of the X_1_X_2_Y type has been cytogenetically identified in six distant *Nothobranchius* spp., which suggests dynamic sex chromosome evolution (Ewulonu et al. 1985; Krysanov et al. 2016; Krysanov and Demidova 2018). Intriguingly, the *N. furzeri* genome sequence revealed an XY sex chromosome pair with polymorphic size of a non-recombining region in different populations (Reichwald et al. 2015; Willemsen et al. 2020). It was hypothesized that the *N. furzeri* Y chromosome polymorphism represents an early stage of sex chromosome evolution (Reichwald et al. 2015). However, physical mapping of various repeats in *N. furzeri* and its sister species *N. kadleci* revealed that repetitive DNA landscape differs considerably between their X and Y chromosomes and these differences extend beyond the non-recombining regions. In particular, compared to their X chromosome counterparts, Y chromosomes possessed largely reduced block of constitutive heterochromatin in the pericentromeric region in two out of three examined populations (Štundlová et al. 2022). This region overlapped with hybridization signals of fluorescence in situ hybridization (FISH) with two satellite repeats, Nfu-SatA and Nfu-SatB, associated with all (peri)centromeric regions in *N. furzeri* and *N. kadleci* (Reichwald et al. 2009; Štundlová et al. 2022).

Satellite DNA (satDNA) is a tandemly repeated DNA class with rapid molecular evolution (Plohl et al. 2012; Garrido-Ramos 2017; Thakur et al. 2021) leading to highly species-specific landscapes differing both quantitatively and qualitatively (Feliciello et al. 2014; Bracewell et al. 2019; Ávila Robledillo et al. 2020). Arrays of satDNA often occupy (peri)centromeric and (sub)telomeric regions where they represent a major component of constitutive heterochromatin (Plohl et al. 2012; Garrido-Ramos 2017), but they may also display non-clustered organization (Ruiz-Ruano et al. 2016; Šatović-Vukšić and Plohl 2023). Certain satDNA repeats can be associated with centromeres (Melters et al. 2013; Hartley and O’Neill 2019; Talbert and Henikoff 2020) and thus are considered to be involved in the segregation of chromosomes during cell divisions (Henikoff et al. 2001; McKinley and Cheeseman 2016). Yet despite their rather conservative function, centromeric satDNAs can have very fast turnover (Henikoff et al. 2001; Bracewell et al. 2019; Ávila Robledillo et al. 2020; Nishihara et al. 2021). It has been hypothesized that this is due to centromere drive (Henikoff et al. 2001), which results from different ability of homologous chromosomes to bind spindle microtubules. Homologs thus can exploit the asymmetric female meiosis producing three polar bodies (i.e., the evolutionary dead-ends) and only one egg, and segregate non-randomly (Henikoff et al. 2001; Kursel and Malik 2018; Kumon and Lampson 2022).

Hence, it was hypothesized that the reduction in (peri)centromeric repeats on Y chromosomes observed in *N. furzeri* and *N. kadleci* reflects relaxed centromere drive (Štundlová et al. 2022), as the Y chromosome never passes through female meiosis (cf. Yoshida and Kitano 2012; Pokorná et al. 2014). Unfortunately, nothing is known about killifish centromeric organization outside *N. furzeri* and *N. kadleci* (Reichwald et al. 2009, 2015; Štundlová et al. 2022), and little is known about the centromere organization in teleost fishes in general. Rather than identifying sequences which bind centromeric proteins (Cech and Peichel 2016; Ichikawa et al. 2017), the available studies have focused mainly on sequences associated with centromeres, detected either by molecular or bioinformatic methods and physically mapped by means of in situ hybridization (Ferreira et al. 2010; Suntronpong et al. 2020; Stornioli et al. 2021; Goes et al. 2022, 2023; Kretschmer et al. 2022). More recently, these sequences have been inferred directly from long read sequencing data (Ichikawa et al. 2017; Conte et al. 2019; Varadharajan et al 2019; Tao et al. 2021). The fish centromeres typically comprise satellite sequences with conserved motifs such as the CENP-B box needed for chromosome stability and cell division (Suntronpong et al. 2016; Gamba and Fachinetti 2020).

In the present study, we analyzed repetitive sequences across the representatives of *Nothobranchius* genus by means of RepeatExplorer2 bioinformatic pipeline (Novák et al. 2020) and focused particularly on the identification of repeats associated with (peri)centromeric regions. Our results suggest that Nfu-SatA and Nfu-SatB, herein designated as NkadSat01-77 and NfurSat01-348, are associated with (peri)centromeres only in one lineage of the Southern clade, although the latter can be detected also in representatives of the Coastal clade. We also identified novel repeat associated with (peri)centromeres in the Coastal-clade species, NrubSat01-48. We discuss rapid evolutionary changes in the distribution of satDNA associated with (peri)centromeric regions and their distinct dynamics in the two *Nothobranchius* clades.

## Materials and methods

### Fish sampling

We analyzed individuals of 14 species representing the Southern and Coastal clade (seven and five species, respectively) of the genus *Nothobranchius*, with *N. ocellatus* and *Fundulosoma thierryi* as their outgroups. The studied individuals from *N. orthonotus*, *N. kuhntae*, *N. pienaari*, *N. rachovii*, *N. eggersi*, and *N. rubripinnis* were sampled from laboratory populations recently derived from wild-caught individuals and were previously identified based on morphology and the phylogenetic analysis of mitochondrial and nuclear DNA markers (for details, see Bartáková et al. 2015; Blažek et al. 2017; Reichard et al. 2022). The remaining species were obtained from specialists and experienced hobby breeders who keep strictly population-specific lineages derived from original imports. In this case, the species identity was confirmed on the basis of key morphological characters (Wildekamp 1996, 2004; Watters et al. 2008, 2020). The detailed information is provided in Table 1.

**Table 1:** List of *Nothobranchius* killifish species used in this study along with their sample sizes (N) and origin

Clade	Species	Code	N*	Source/locality
Outgroup	*Fundulosoma thierryi* Ahl, 1924	FTH	1♂, 3♀	Aquarium strain
Southern	***Nothobranchius furzeri* Jubb, 1971**	**NFU**	**2♂, 1♀**	**Chefu, MZ**
	***N. kadleci* Reichard, 2010**	**NKA**	**1♂, 1♀**	**Gorongosa, MZ**
	***N. orthonotus* (Peters, 1844)**	**NOR**	**3♂, 3♀**	**Limpopo, MZ**
	*N. kuhntae* (Ahl, 1926)	NKU	4♂, 3♀	Pungwe, MZ
	*N. pienaari* Shidlovskyi, Watters & Wildekamp, 2010	NPI	2♂, 3♀	Limpopo, MZ
	*N. krysanovi* Shidlovskyi, Watters & Wildekamp, 2010	NKR	2♂, 2♀	Quelimane, MZ
	***N. rachovii* Ahl, 1926**	**NRA**	**2♂, 2♀**	**Beira Airport, MZ**
Ocellatus (outgroup)	*N. ocellatus* (Seegers, 1985)	NOC	1♂, 1♀	Nyamwage, TZ
Coastal	*N. eggersi* Seegers, 1982	NEG	2♂, 1♀	Bagamoyo, TZ
	*N. foerschi* Wildekamp & Berkenkamp, 1979	NFO	2♂	Soga, TZ
	***N. guentheri* (Pfeffer, 1983)**	**NGU**	**4♂, 3♀**	**Zanzibar, TZ**
	*N. cardinalis* Watters, Cooper & Wildekamp, 2008	NCA	1♂	Matandu, TZ
	***N. rubripinnis* Seegers, 1986**	**NRU**	**2♂, 2♀**	**Kitonga, TZ**

*number and sex of individuals used for each method is specified in Supplementary Table 1

Species used for bioinformatic analysis of repeats are in bold. Species order reflects their phylogenetic relationships (for details, see Fig. 2)

### Chromosomal preparations

Mitotic chromosome spreads were obtained either (i) from regenerating caudal fin tissue (Völker and Ráb 2015) with modification described in Sember et al. (2015) and a fin regeneration time ranging from one to two weeks or (ii) by a direct preparation from the cephalic kidney following Ráb and Roth (1988) and Kligerman and Bloom (1977), with the latter protocol being modified according to Krysanov and Demidova (2018). In the kidney-derived preparations, the chromosomal spreading quality was enhanced using a dropping technique by Bertollo et al. (2015). Preparations were inspected with phase-contrast optics and those of sufficient quality were dehydrated in an ethanol series (70%, 80%, and 96%, 2 min each) and stored at − 20 °C until use.

### Constitutive heterochromatin staining

Analysis of constitutive heterochromatin distribution was done by C-banding (Haaf and Schmid 1984), using 4′,6-diamidino-2-phenolindole (DAPI) (1.5 µg/mL in anti-fade; Cambio, Cambridge, UK) counterstaining. The size of heterochromatin blocks was evaluated based on visual comparisons between species. Fluorescent staining with the GC-specific fluorochrome Chromomycin A_3_ (CMA_3_) and the AT-specific fluorochrome DAPI (both Sigma-Aldrich, St. Louis, MO, USA) was performed according to Mayr et al. (1985) and Sola et al. (1992).

### Whole-genome sequencing data

Genomic DNA was sequenced de novo in *Nothobranchius guentheri*, *N. kadleci*, *N. orthonotus*, *N. rachovii*, and *N. rubripinnis*. First, high molecular weight genomic DNA (HMW gDNA) was extracted from three females of each species using the MagAttract HMW DNA Kit (Qiagen, Hilden, Germany), following the provided protocol. Next, Illumina paired-end libraries with 450 bp insert size and 150 bp read length were prepared from the isolated HMW gDNA and sequenced on the NovaSeq 6000 platform at Novogene (HK) Co., Ltd. (Hong Kong, China), yielding, at least, 5 Gb (ca 3.3 × coverage of *Nothobranchius furzeri* genome; 1C = 1.54 Gb, Reichwald et al. 2015; Willemsen et al. 2020). Resulting data were deposited into the Sequence Read Archive (SRA) under the BioProject accession no. PRJNA991117. *N. furzeri*, sequencing data from three female specimens were obtained from the SRA (accession no. ERR583470, ERR583471, and SRR1261480; Reichwald et al. 2015).

### Analysis of repetitive DNA

The collection of satDNA repeats (referred to as satellitome; Ruiz-Ruano et al. 2016) was characterized using RepeatExplorer2 (Novák et al. 2020). Prior to the analysis, the quality of raw Illumina reads was checked using FastQC (version 0.11.5; Andrews 2010). Low quality reads and adapter sequences were removed using cutadapt (version 1.15; Martin 2011) with settings for two-color chemistry: ‘–nextseq-trim = 20 -u -50 -U -50 -m 100 -a AATGATACGGCGACCACCGAGATCTACACTCTTTCCCTACACGACGCTCTTCCGATCT -A GATCGGAAGAGCACACGTCTGAACTCCAGTCACNNNNNNATCTCGTATGCCGTCTTCTGCTTG.’ For comparative analysis, 800,000 read pairs (ca 0.1 × genome coverage of *N. furzeri*) were pseudorandomly subsampled from each biological replica of each species with the RepeatExplorer2 sampleFasta.sh script, using different seed numbers. Resulting subsets were concatenated and analyzed together. The RepeatExplorer2 pipeline was run on the Galaxy server (The Galaxy Community 2022) with Metazoa version 3.0 protein domain database and automatic filtering of abundant repeats. In addition, the repeats were studied in each species independently, using a set of 7,125,000 reads (ca 0.5 × coverage) and equivalent RepeatExplorer2 parameters. Calculation of G + C content and reciprocal BLAST were performed in GeneiousPrime (version 2020.1.2; https://www.geneious.com). To target potential repeats associated with (peri)centromeric regions, the results of the single-species analysis were confined to high confidence satellites with estimated abundance in the genome at least 0.15% and monomer length < 1 kb only. The satellites were named following the nomenclature rules by Ruiz-Ruano et al. (2016), i.e., satDNA name begins with the species abbreviation, followed by the term “Sat,” number reflecting the order of decreasing satDNA abundance in the genome and consensus monomer length. In case of shared satellites, the name of the satDNA was selected according to the species in which it presented the highest abundance in the comparative analysis.

### Identification of putative CENP-B box

SatDNAs with confirmed centromeric localization (see below) were manually inspected for the presence of 17-bp-long CENP-B box motif (Suntronpong et al. 2016) using Geneious Prime. Alignment of putative CENP-B sequences from human (*Homo sapiens*; Masumoto et al. 1989), threespine stickleback (*Gasterosteus aculeatus*; Cech and Peichel 2015), ninespine stickleback (*Pungitius pungitius;* Varadharajan et al. 2019), Asian swamp eel (*Monopterus albus;* Suntronpong et al. 2020), and turquoise killifish (*Nothobranchius furzeri*; this work) were performed with a Geneious native algorithm with default settings for global alignment.

### Fluorescence in situ hybridization (FISH)

#### Preparation of FISH probes

We previously characterized Nfu-SatA (here designated as NkadSat01-77) and Nfu-SatB (NfurSat01-348) as the most abundant satellite repeats in *N. furzeri* and *N. kadleci*, respectively (Štundlová et al. 2022). FISH probe covering the whole monomer length (77 bp) of NkadSat01-77 was generated as an oligonucleotide labeled with Cy3 at its 5′ end (Generi Biotech, Hradec Králové, Czech Republic). The same applies also to other probes for repeats with a short monomer unit (< 100 bp) characterized for the first time in this study, i.e., NrubSat01-48, NfurSat02-39, NkadSat02-76, and NgueSat01-63 (see Table 2). In the case of NfurSat01-348 with 348-bp-long monomer, the PCR-amplified fragments have beed cloned and verified previously (Štundlová et al. 2022), and clones containing a trimer of NfurSat01-348 were used for construction of the FISH probes. The entire plasmids were labeled by nick translation using a Cy3 NT Labeling Kit (Jena Bioscience, Jena, Germany). For the final hybridization mix, 250–500 ng of the labeled plasmid and 12.5–25 µg of sonicated salmon sperm DNA (Sigma-Aldrich) were applied per slide. The final hybridization mixtures for each slide (15 µL) were prepared according to Sember et al. (2015).

**Table 2:** High confidence satellites identified by comparative analysis with RepeatExplorer2

Satellite	Monomer (bp)	GC (%)	Avg reads per replica						Notes
			NFU	NKA	NOR	NRA	NGU	NRU	
**NfurSat01-348**	**348**	**41.1**	**2933**	**1508**	**643**	**50**	**23**	**9**	Previously identified Nfu-SatB*
**NkadSat01-77**	**77**	**63.6**	**560**	**2533**	**369**	**0**	**2**	**0**	Previously identified Nfu-SatA*
NortSat01-169	169	36.1	466	164	4700	34	18	50	
**NrubSat01-48**	**48**	**45.8**	**0**	**0**	**0**	**0**	**195**	**2107**	**Specific for Coastal clade**
NkadSat03-93	93	60.2	398	1240	262	42	521	233	
**NfurSat02-39**	**39**	**20.5**	**1160**	**836**	**19**	**21**	**14**	**28**	**High abundance in *N. furzeri* and *N. kadleci***
NrubSat02-394	394	60.2	62	313	119	108	33	996	
NfurSat03-49	49	65.3	1261	217	30	0	0	0	
NfurSat04-84	84	31	763	214	86	207	178	68	
NfurSat05-24	24	58.3	556	27	3	2	13	7	
**NkadSat02-76**	**76**	**57.9**	**0**	**512**	**2**	**0**	**0**	**0**	**Similarity hits with NkadSat01-77**
NgueSat02-662	662	46.8	1	0	10	163	266	2	
NfurSat06-21	21	19	304	20	56	14	17	18	
NortSat02-20	20	45	0	0	356	0	1	4	
**NgueSat01-63**	**63**	**31.7**	**0**	**0**	**0**	**0**	**193**	**137**	**Specific for Coastal clade**
NfurSat07-691	691	42.1	87	46	67	45	35	37	
NrubSat03-976	976	40.1	47	31	40	48	41	61	
NfurSat08-980	980	37.3	49	42	29	37	29	41	
NortSat03-67	67	43.3	0	0	208	0	0	0	
NfurSat09-909	909	36.9	39	27	29	20	39	39	
NrubSat04-490	490	44.3	27	15	16	29	33	44	

*Reichwald et al. 2009; Štundlová et al. 2022

Markers selected for physical mapping are indicated in bold

#### Standard FISH analysis

Single-color FISH experiments with NfurSat01-348 probe were carried out following Sember et al. (2015) (slide pre-treatment, probe/chromosomes denaturation, and hybridization conditions) and Yano et al. (2017) (post-hybridization washing), with modifications described in Štundlová et al. (2022). Briefly, following the standard pre-treatment steps, chromosomes were denatured in 75% formamide in 2 × SSC (pH 7.0) (Sigma-Aldrich) at 72 °C for 3 min. The hybridization mixture was denatured at 86 °C for 6 min. The hybridization took place overnight (17–24 h) at 37 °C in a moist chamber. Subsequently, non-specific hybridization was removed twice in 1 × SSC (pH 7.0) (65 °C, 5 min each) and once in 4 × SSC in 0.01% Tween 20 (42 °C, 5 min), followed by washing in 1 × PBS (1 min at room temperature; RT). Slides were dehydrated in an ethanol series (70%, 80%, and 96%, 2 min each) and then mounted in anti-fade containing 1.5 µg/mL DAPI (Cambio, Cambridge, UK).

#### *Non-denaturing FISH* (*ND-FISH*)

Remaining five satDNA probes, i.e., 5′-end-labeled oligonucleotides (NkadSat01-77, NrubSat01-48, NfurSat02-39, NkadSat02-76, and NgueSat01-63; more details provided below in Results section and Table 2) were mapped using ND-FISH according to Cuadrado and Jouve (2010) with some modifications. Briefly, a total of 30 µL of hybridization mixture containing 2 pmol/µL of labeled oligonucleotides in 2 × SSC were used per slide. Then, the mixture was denatured at 80 °C for 5 min and immediately placed on ice. After that, the denatured hybridization mixture was transferred onto the slides with neither pre-treatment steps nor chromosome denaturation. After 2 h of hybridization at RT, the slides were washed with 4 × SSC 0.2% Tween 20 for 10 min, followed by 5 min washing in 4 × SSC 0.1% Tween 20 (both at RT and shaking). Chromosome preparations were then passed through ethanol series (70%, 80%, and 96%, 3 min each) and then air dried. Chromosomes were counterstained with 20 µL of DABCO anti-fade (1,4-diazabicyclo(2.2.2)-octane) containing 0.2 µg/mL DAPI (both Sigma-Aldrich) or in anti-fade containing 1.5 µg/mL DAPI Cambio, Cambridge, UK).

### Microscopic analyses and image processing

Images from all cytogenetic methods were captured using a BX53 Olympus microscope equipped with an appropriate fluorescence filter set and coupled with a black and white CCD camera (DP30W Olympus). Images were acquired for each fluorescent dye separately using DP Manager imaging software (Olympus), which was further used also to superimpose the digital images with the pseudocolors (red for CMA_3_ and green for DAPI in case of fluorescence staining; blue for DAPI and red for Cy3 in case of FISH). Composite images were then optimized and arranged using Adobe Photoshop, version CS6.

At least 20 chromosome spreads per individual and method were analyzed. Chromosomes were classified according to Levan et al. (1964) but modified as m – metacentric, sm – submetacentric, st – subtelocentric, and a – acrocentric, where st and a chromosomes were scored together into st-a category.

## Results

### Previous basic karyotype characteristics confirmed

Individuals from all studied species displayed mostly the same 2n and highly similar proportion of chromosome categories as previously reported (Reichwald et al. 2009, 2015; Krysanov and Demidova 2018; Štundlová et al. 2022; Lukšíková et al. 2023). The only exception was *N. ocellatus*, where we recorded 2n = 32 with the karyotype being composed exclusively of monoarmed (st-a) chromosomes, in contrast to previously reported 2n = 30 with one chromosome pair being large metacentric (Krysanov and Demidova 2018). The individuals studied by Krysanov and Demidova (2018) were later found to be members of a newly described closely related species *N. matanduensis* (Watters et al. 2020) (S. Simanovsky, pers. commun.). Finally, in line with the previous reports (Ewulonu et al. 1985; Krysanov and Demidova 2018; Lukšíková et al. 2023), *Fundulosoma thierryi* and *N. guentheri* possessed male heterogametic X_1_X_1_X_2_X_2_/X_1_X_2_Y multiple sex chromosome system manifested by different chromosome counts between males and females (males had one chromosome less) and particularly in *N. guentheri* the male-limited neo-Y chromosome was discernible as the only large sm/st element in the complement.

### High interspecific variability in distribution and composition of constitutive heterochromatin

**Fig. 1:**
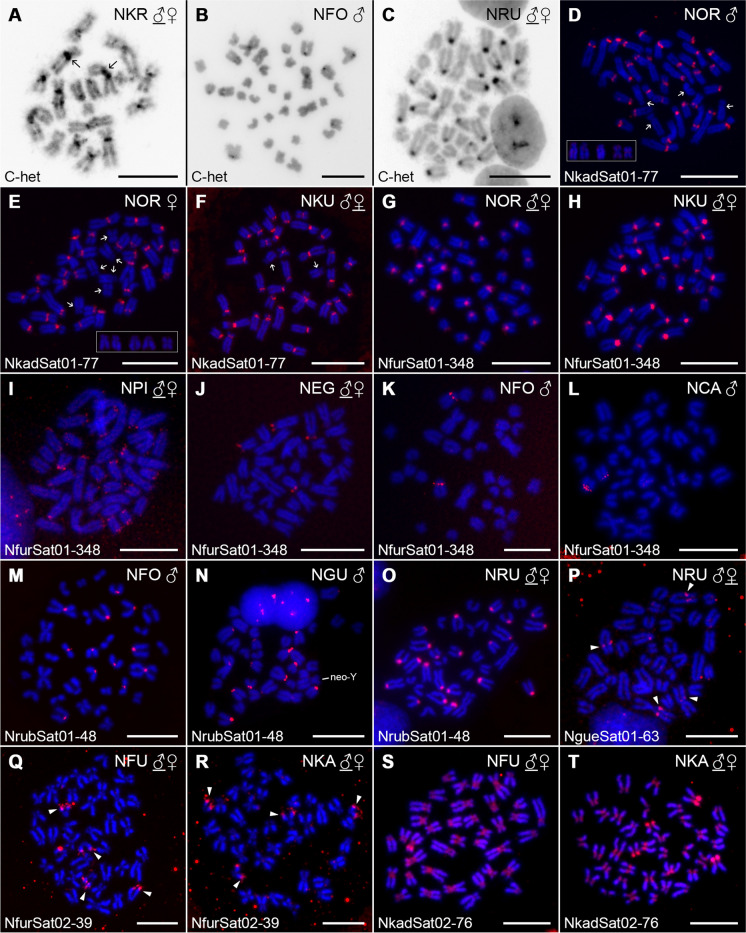
Selected representative mitotic metaphases of studied *Nothobranchius* species after C-banding and FISH with satDNA probes. A full set of results from all studied species is provided in Supplementary Figs. 1, 5, 6, 7, and 8. (**A**–**C**) C-banding. Arrows indicate examples of huge pericentromeric heterochromatin blocks in expected fusion sites on large metacentric chromosomes of *N. krysanovi* (**A**). Note: differences between constitutive heterochromatin amount and distribution between Southern-clade (**A**) and Coastal-clade species *N. foerschi* (**B**) and *N. rubripinnis* (**C**). (**D**–**T**) FISH with satDNA repeats (red signals) in species with positive results. Sex of the studied individuals is indicated and eventually underlined where both sexes (if studied) presented the same distribution pattern (i.e., except for *N. orthonotus*; **D**, **E**). In the case of NkadSat01-77 repeat in *N. orthonotus* (**D**, **E**) and *N. kuhntae* (**F**), arrows point to chromosomes lacking the (peri)centromeric signals. Polymorphic patterns regarding this feature are framed. Neo-Y chromosome in *N. guentheri* male (**N**) can be identified based on distinctive morphology. For better clarity, arrowheads point on signals after FISH with NgueSat01-63 (**P**) and NfurSat02-39 (**Q**, **R**) probes. Species acronyms are summarized in Table 1. Chromosomes were counterstained with DAPI (blue). Scale bar = 10 µm

Amount of constitutive heterochromatin varied among the studied *Nothobranchius* spp. (Fig. 1A–C; Supplementary Fig. 1). Within the chromosome complements of *N. cardinalis*, *N. guentheri*, and *N. rubripinnis*, the largest metacentric chromosome pair either lacked or had unremarkable/notably smaller C-bands compared to the remainder of the chromosome set (Fig. 1C; Supplementary Fig. 1J–M). In *N. foerschi*, the largest metacentric chromosome pair possessed a distinct pericentromeric C-band, while the second largest metacentric pair displayed only tiny heterochromatin block (Fig. 1B; Supplementary Fig. 1I). By contrast, majority of large biarmed chromosomes in species of the Southern clade possessed large heterochromatin segments (e.g., Fig. 1A; more details provided below). In addition to pericentromeric bands, heterochromatin accumulations were present on the short arms of several chromosomes in *N. eggersi*. In males of *N. guentheri*, neo-Y sex chromosome bore an apparent C-banded region on its long arms (Supplementary Fig. 1J, arrowhead). The other species with known X_1_X_2_Y multiple sex chromosome system (*F. thierryi*) did not show any exceptional C-banding pattern on these sex chromosomes. Four st-a chromosomes in *F. thierryi* displayed remarkable heterochromatin blocks covering their short arms. In the Southern clade, *N. orthonotus* and *N. kuhntae* featured the highest amount and diversity of heterochromatin blocks which were distributed on multiple regions across the chromosome complement. This observation is consistent with large (peri)centromeric regions found previously in closely related *N. furzeri* and *N. kadleci* (Štundlová et al. 2022; see Supplementary Fig. 2A, B for comparison). On the other hand, chromosomes of *N. pienaari*, *N. krysanovi*, and *N. rachovii* bore almost exclusively pericentromeric bands of variable lengths, some of them being remarkably large (Fig. 1A; Supplementary Fig. 1D–F). In the species with almost exclusively biarmed (metacentric or submetacentric) chromosomes and low 2n, namely *N. krysanovi* and *N. rachovii*, some (peri)centromeres were arranged as two large adjacent blocks. *N. krysanovi* also displayed additional interstitial heterochromatin blocks on several chromosomes. In *N. rachovii*, only two large submetacentric chromosomes possessed very tiny interstitial bands in addition to pericentromeric ones.

Fluorescent staining revealed, besides few predominantly DAPI^+^ (AT-rich) bands (e.g., in *F. thierryi*, *N. orthonotus*), variable amount and distribution of CMA_3_^+^ (GC-rich) regions. Five species (*F. thierryi*, *N. pienaari*, *N. krysanovi*, and *N. foerschi*) displayed just one pair of clear terminal or interstitial signals, highly likely overlapping with major ribosomal DNA (rDNA) cluster (cf. Sember et al. 2015 and references therein). Similar signals were revealed also on the neo-Y and at least one X chromosome of *N. guentheri* (Supplementary Fig. 3J). Several *N. guentheri* chromosomes also featured additional tiny (peri)centromeric signals on at least four chromosomes (Supplementary Fig. 3J, K). In *N. rachovii*, terminal CMA_3_^+^ signals were observed on the short arms of the smallest acrocentric chromosome pair, and at least four large metacentrics/submetacentrics had tiny centromeric signals (Supplementary Fig. 3F). *N. ocellatus* and *N. eggersi* bore up to seven and up to four signals, respectively (Supplementary Fig. 3G, H). *N. cardinalis* and *N. rubripinnis* shared the CMA_3_ pattern in the way that (peri)centromeres of all chromosomes were GC-rich except for the one pair of large metacentric chromosomes (Supplementary Fig. 3L, M). Finally, almost all chromosome pairs in *N. orthonotus* and *N. kuhntae* had GC-rich (peri)centromeres (Supplementary Fig. 3B, C), similarly to patterns found in *N. furzeri* and *N. kadleci* (Štundlová et al. 2022; Supplementary Fig. 2C, D).

### RepeatExplorer2 reveals candidate (peri)centromeric repeats with different abundances among species

The comparative analysis of tandem repeats in representatives of the Southern (*N. furzeri*, *N. kadleci*, *N. orthonotus*, *N. rachovii*) and Coastal (*N. guentheri*, *N. rubripinnis*) clades revealed in total 21 high confidence satellites with various abundances (Table 2). The two most abundant tandem repeats, namely NfurSat01-348 and NkadSat01-77, were the previously studied putative centromeric repeats Nfu-SatB and Nfu-SatA, respectively. Besides *N. furzeri* and *N. kadleci*, these clusters were also abundant in *N. orthonotus*. Similar pattern was observed for a less abundant repeat, NfurSat02-39, which was also present in high amounts in *N. furzeri* and *N. kadleci*, but much less in the other species. In addition, we found a sequence similarity between the abovementioned NkadSat01-77 repeat and NkadSat02-76 (pairwise identity 84.7%), which contained majority of reads only from *N. kadleci* (Table 3). Notably, all these satellites showed limited occurence or were missing in *N. rachovii*, *N. guentheri*, and *N. rubripinnis*, suggesting existence of different sequences in (peri)centromeres of these species. Indeed, satellites NrubSat01-48 and NgueSat01-63 showed the opposite pattern, as they were present in *N. rubripinnis* and *N. guentheri* but missing in the rest of the surveyed species. Single-species analysis with more stringent criteria (estimated abundance in the genome at least 0.15% and monomer length < 1 kb) confirmed these results.

**Table 3:** Species-specific analysis of the most abundant satellites (abundance > 0.15% of the genome, monomer length < 1 kb)

Species	Cluster	Satellite ID (from comparative analysis)	Estimated genome proportion (%)	Notes
*N. furzeri*	**NFU-1**	**NfurSat01-348**	**16**	Previously identified Nfu-SatB*
	**NFU-12**	**NkadSat01-77**	**3.5**	Previously identified Nfu-SatA*
	**NFU-7**	**NfurSat02-39**	**0.48**	
	NFU-8	NfurSat03-49	0.47	
	NFU-29	NfurSat05-24	0.27	
	NFU-53	NfurSat05-24	0.2	
	NFU-83	NkadSat03-93	0.15	
*N. kadleci*	**NKA-1**	**NkadSat01-77**	**9.6**	Previously identified Nfu-SatA*
	**NKA-2**	**NfurSat01-348**	**7.5**	Previously identified Nfu-SatB*
	NKA-15	NkadSat03-93	0.48	
	**NKA-34**	**NfurSat02-39**	**0.34**	
	NKA-67	**-**	0.22	
	**NKA-73**	**NkadSat02-76**	**0.2**	
	NKA-83	**-**	0.19	
*N. orthonotus*	**NOR-59**	**NfurSat01-348**	**2.3**	Previously identified Nfu-SatB*
	NOR-1	NortSat01-169	2.2	
	**NOR-24**	**NkadSat01-77**	**0.41**	Previously identified Nfu-SatA*
*N. rachovii*	No satellites fitting the criteria were identified			
*N. guentheri*	**NGU-94**	**NrubSat01-48**	**0.18**	
*N. rubripinnis*	**NRU-1**	**NrubSat01-48**	**2**	
	NRU-23	NrubSat02-394	0.37	

*Reichwald et al. 2009; Štundlová et al. 2022

Markers selected for physical mapping are indicated in bold

Intriguingly, we found a putative CENP-B box sequence in NfurSat01-348 repeat. Alignment to human CENP-B box sequence showed 0.47 identity, which is similar to other fish species (Supplementary Fig. 4). This finding along with the length of NfurSat01-348 monomer (348 bp; i.e., approx. twice the length of the nucleosome unit) may imply a possible role of NfurSat01-348 in centromere function (Talbert and Henikoff 2020). However, no CENP-B box related motif was recovered in other inspected repeats, including the NrubSat01-48 (peri)centromeric satellite of *N. rubripinis.*

### Physical mapping of six candidate (peri)centromeric satDNA monomers shows different patterns at intra- and inter-clade levels

FISH with NkadSat01-77 probe revealed detectable clusters only in *N. orthonotus* and *N. kuhntae* (Fig. 1D–F; Supplementary Fig. 5B–D). All signals were restricted to (peri)centromeric regions of almost all chromosomes, corroborating patterns found in *N. furzeri* and *N. kadleci* (Štundlová et al. 2022; Supplementary Fig. 2E, F). While all *N. kuhntae* individuals shared the same pattern (i.e., all but one chromosome pair carrying the signal; Fig. 1F; Supplementary Fig. 5D), individuals of *N. orthonotus* displayed site-number variability, with the number of chromosomes lacking the signal being either four (1 male), five (1 male, 1 female), or six (2 males, 1 female) chromosomes (Fig. 1D, E; Supplementary Fig. 5B–C).

Detectable clusters of NfurSat01-348 were found in (peri)centromeric regions of all chromosomes in *N. orthonotus*, *N. kuhntae* (i.e., the same pattern as in *N. furzeri* and *N. kadleci*; Štundlová et al. 2022 and Supplementary Fig. 2G, H), and in (peri)centromeric or terminal regions of about one-third of the chromosome complement in *N. pienaari* (Fig. 1G–I; Supplementary Fig. 6B–D). Besides these species of Southern clade, we also found clear hybridization patterns in three species of Coastal clade. Individuals of *N. eggersi* showed four signals placed terminally on short arms of st-a chromosomes (Fig. 1J; Supplementary Fig. 6H), *N. foerschi* and *N. cardinalis* each carried one pair of st-a chromosomes with (peri)centromeric signals (Fig. 1K, L; Supplementary Fig. 6I, K). The pair was small-sized in *N. cardinalis* and among the largest in *N. foerschi*. The NfurSat01-348 loci in *N. foerschi* coincided with CMA_3_^+^ sites (compare Supplementary Figs. 3I and 6I).

Satellite repeat NrubSat01-48 was detected only in three species of Coastal clade: *N. rubripinnis* (from which it was isolated), *N. foerschi*, and *N. guentheri* (Fig. 1M–O; Supplementary Fig. 7K, L, N). The repeat clusters were located exclusively in the (peri)centromeric regions, but none of the mentioned species possessed them in all chromosomes. Studied *N. foerschi* and *N. guentheri* males displayed 12 and 16 signals, respectively (Fig. 1M, N; Supplementary Fig. 7K, L). In *N. rubripinnis*, 22 out of 36 chromosomes bore the signal (Fig. 1O; Supplementary Fig. 7N).

The second satellite limited to *N. rubripinnis* and *N. guentheri* (NgueSat01-63) was hybridized in both these species, however, signals were detected only on the long arms of four chromosomes in *N. rubripinnis* (Fig. 1P; Supplementary Fig. 8A, B)*.* The lack of signal in *N. guentheri* could be explained either by its abundance being below the FISH detection threshold, or by different organization of this repeat in the genome.

NfurSat02-39, shared by *N. furzeri* and *N. kadleci,* was present in both sexes of these species, but no positive FISH signals were observed in *N. orthonotus* (Fig. 1Q, R; Supplementary Fig. 8C–E)*.* In both species, signals were localized in the long arms of two pairs of chromosomes in both males and females.

The last hybridized marker was NkadSat02-76, bearing similarity hits with NkadSat01-77. Positive signals from this satDNA were observed in all centromeres in both sexes of *N. furzeri* and *N. kadleci*. The only difference in the signal pattern between these two species was related to additional prominent signals located terminally on the short arms of two (*N. furzeri*) and four (*N. kadleci*) chromosomes, respectively (Fig. 1S, T; Supplementary Fig. 8F, G).

**Fig. 2:**
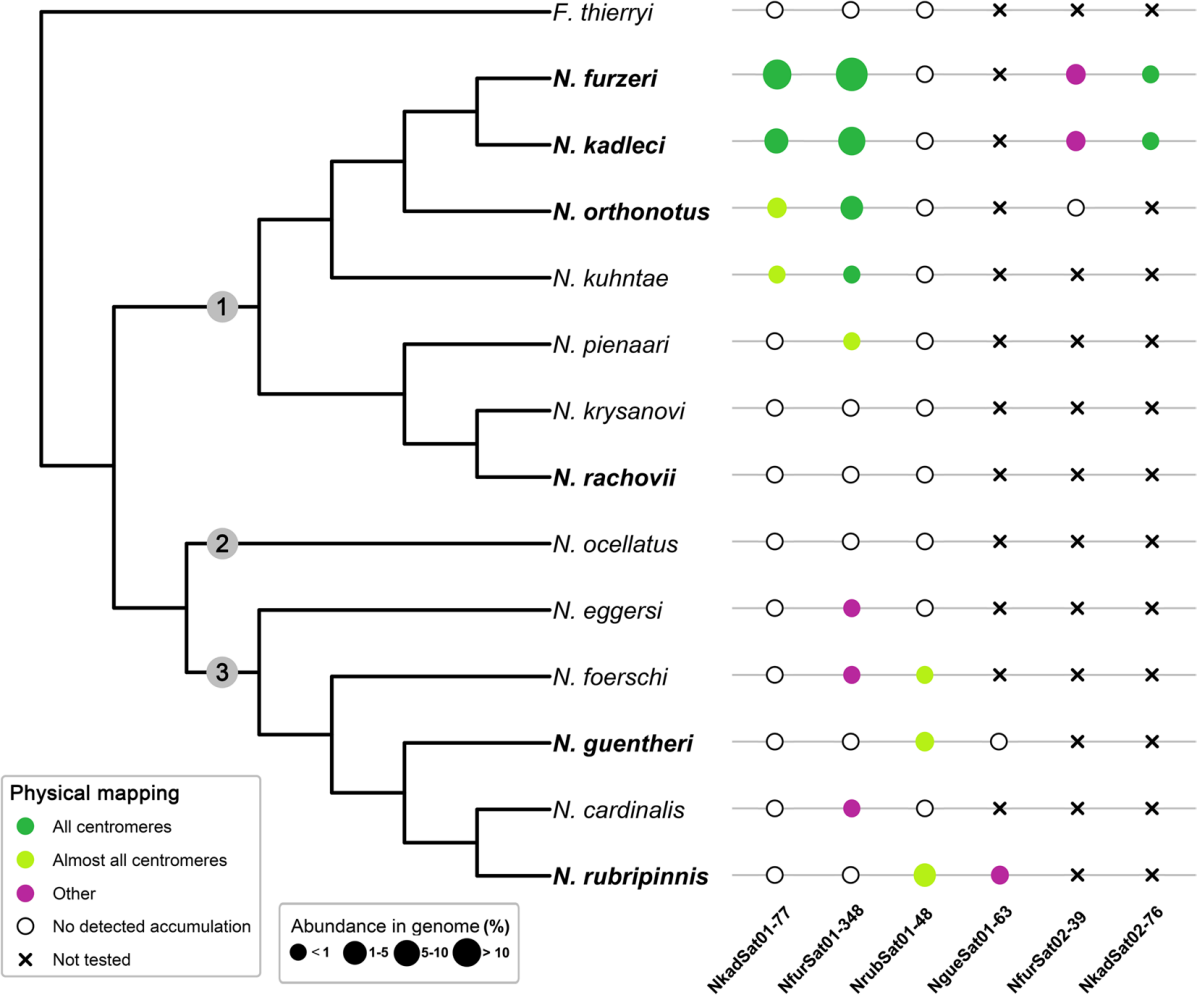
Phylogenetic relationships and patterns of selected satDNA monomers in inspected *Nothobranchius* species. Simplified phylogenetic tree is based on van der Merwe et al. (2021). The phylogenetic positions of *N. kadleci* and *N. kuhntae* were inferred from Dorn et al. (2014). Colored circles represent positive FISH signals in different chromosomal locations. The size of the circles reflects the abundance in the genome for respective satDNA. Abundance in the genome (%) is set as ranges. Lack of positive signals after FISH is demarcated by empty circles. Black crosses indicate that a given satDNA was not physically mapped in the particular species. Note that abundance in the genome might not perfectly correlate with chromosomal distribution revealed by physical mapping because some portion of respective tandem repeats may be present in low-copy clusters undetectable by FISH. Species which were subject to RepeatExplorer2 analysis are shown in bold. Numbers in grey circles in the phylogenetic tree denote distinct *Nothobranchius* clades: (1) Southern; (2) Ocellatus; (3) Coastal

A summary of patterns of satDNA monomers selected for chromosomal mapping is provided in Fig. 2.

## Discussion

During the last decade, the satDNA research has been greatly boosted by implementation of bioinformatic pipelines allowing for de novo repeat identification in low-coverage sequencing data (Garrido-Ramos 2017; Lower et al. 2018; Novák et al. 2020; Vondrak et al. 2020; Šatović-Vukšić and Plohl 2023). An increasing number of studies compare satellitomes of closely related species in diverse taxonomic groups (e.g. Pita et al. 2017; Palacios-Gimenez et al. 2020; de Lima and Ruiz-Ruano 2022; Despot-Slade et al. 2022; Peona et al. 2022; Mora et al. 2023) including fishes (de Silva et al. 2017; Goes et al. 2022; Kretschmer et al. 2022) and thereby provide a thorough insight into pace and mechanisms of satDNA evolution.

In the present study, we performed comparative cytogenetic and bioinformatic analyses of satellite DNA across the species of Southern and Coastal clade of the killifish genus *Nothobranchius* to reveal dynamics of repeats associated with (peri)centromeric regions.

Our results showed the presence of extended C-banded pericentromeric heterochromatin regions in some of the large metacentric chromosomes of *N. pienaari*, *N. krysanovi*, and *N. rachovii* (Fig. 1A; Supplementary Fig. 1D–F). It is consistent with our previous findings in the remaining Southern-clade species, *N. furzeri* and *N. kadleci* (Štundlová et al. 2022 and Supplementary Fig. 2A, B) where we reported large amounts of pericentromeric heterochromatin in almost all chromosomes of the complement. By contrast, our present study shows that with the sole exception of one chromosome pair in *N. foerschi*, large metacentric chromosomes originating from fusions either lacked or had notably smaller C-bands than other chromosomes in the Coastal-clade species *N. cardinalis*, *N. foerschi*, *N. guentheri*, and *N. rubripinnis* (e.g., Fig. 1B, C)*.* These findings indicate differences in mechanisms underpinning karyotype change between Southern-clade and Coastal-clade killifishes. The interspecific variability in amount and distribution of constitutive heterochromatin found herein in *Nothobranchius* spp. is analogous to patterns found previously in several other fish groups, including *Chromaphyosemion* killifishes (Völker et al. 2008), gobiid fishes (Caputo et al. 1997), and loricariids (Ziemniczak et al. 2012).

We performed RepeatExplorer2 analysis in *N. guentheri*, *N. kadleci*, *N. orthonotus*, *N. rachovii*, and *N. rubripinnis* and included also data available for the model species *N. furzeri*. In total, the RepeatExplorer2 comparative analysis revealed 21 satellite sequences. The most abundant of them (NkadSat01-77, NfurSat01-348, NrubSat01-48, and NfurSat02-39) and two additional satellites (NkadSat02-76 sharing similary with NkadSat01-77, and NgueSat01-63 specific for the Coastal clade) were physically mapped across both clades and the outgroups by FISH.

Štundlová et al. (2022) reported two satellites, Nfu-SatA and Nfu-SatB (previously identified also in other *N. furzeri* strains; Reichwald et al. 2009, 2015), to be the most abundant repeat types in both the *N. furzeri* and *N. kadleci* genomes. Both repeats herein designated as NkadSat01-77 and NfurSat01-348 were mapped to pericentromeric constitutive heterochromatin blocks of varying sizes in these two sister species (see also Supplementary Fig. 2E–H). Our results suggest that NkadSat01-77 is restricted to the *N. furzeri* lineage as it is present, albeit in lower abundance, also in (peri)centromeric regions of almost all chromosomes in *N. orthonotus* and *N. kuhntae* (Fig. 1D–F; Supplementary Fig. 5B–D). Inter-individual variability in the number of pericentromeric signals was observed in *N. orthonotus* but not in *N. kuhntae* and was not related to sex. This points to possible differences in the dynamics of gradual repeat change between the two closely related species. Furthermore, the satellite NkadSat02-76 was detected in the (peri)centromeric regions of all chromosomes in *N. furzeri* and *N. kadleci* only (Fig. 1S, T; Supplementary Fig. 8F, G)*.* Given that this repeat is specific for *N. kadleci* genome (Table 2), it likely represents a new sequence variant of NkadSat01-77. Their high sequence similarity was apparently responsible for observing a positive NkadSat01-77 hybridization also in (peri)centromeres of *N. furzeri*. Another highly abundant satDNA, NfurSat01-348, was also detected in (peri)centromeric regions of all chromosomes in *N. orthonotus* and *N. kuhntae* (Fig. 1G, H; Supplementary Fig. 6B, C)*.* It was further present in detectable amounts also in *N. pienaari* as well as *N. eggersi*, *N. foerschi*, and *N. cardinalis* of the Coastal clade (Fig. 1I–L; Supplementary Fig. 6D, H, I, K). The NfurSat01-348 signals were located terminally on the short arms of two chromosome pairs in *N. eggersi*, while they resided in (peri)centromeric regions of about one third of the complement in *N. pienaari* and one chromosome pair of each *N. foerschi* and *N. cardinalis*. NfurSat01-348 thus seems to be shared across *Nothobranchius* spp. but it got amplified and associated with (peri)centromeres in the *N. furzeri* lineage of the Southern clade.

While NkadSat01-77 and NfurSat01-348 were restricted to Southern clade species, satellites NrubSat01-48 and NgueSat01-63 mirrored this pattern as they were detected in the Coastal clade only. The NgueSat01-63 was localized on only four chromosomes in *N. rubripinnis* (Fig. 1P) and could not have been detected by FISH on chromosomes of *N. guentheri* (Supplementary Fig. 8A). However, NrubSat01-48 was successfully mapped in three representatives of the Coastal clade (Fig. 1M–O; Supplementary Fig. 7 K, L, N). The hybridization signals were detected exclusively in the (peri)centromeric regions of majority but not all chromosomes in *N. rubripinnis, N. foerschi*, and *N. guentheri.* Corroborating the C- and fluorescent-banding patterns, NrubSat01-48 clusters were absent in (peri)centromeres of some large metacentric chromosomes (Fig. 1M, O; Supplementary Fig. 7K, N).

Interestingly, none of the above tested satellites was detected in (peri)centromeres of *N. rachovii* (Supplementary Figs. 5G, 6F, 7H), and the RepeatExplorer2 analysis failed to identify any satellites potentially associated with centromeres in this species (Tab. 2, Tab. 3). Blocks of pericentromeric heterochromatin visible on most *N. rachovii* chromosomes (Supplementary Fig. 1F) suggest the presence of tandem arrays. A possible explanation might be a presence of microsatellites as centromeric localization of short repeats has been reported in various organisms (e.g., Kim et al. 2002; Chang et al. 2008) and their presence could escape RepeatExplorer2 analysis as this tool is known to omit low complexity sequences (Novák et al. 2020).

In both *N. furzeri* and *N. kadleci*, the large blocks of pericentromeric heterochromatin coincide with higher numbers of chromosome arms but not with different number of chromosomes than expected when compared to karyotypes of other *Nothobranchius* spp. (Krysanov and Demidova 2018). It suggests that evolution of satellite DNA in *Nothobranchius* species is associated either with intrachromosomal rearrangements or centromere repositioning, i.e., inactivation of an existing centromere and de novo formation of a new one elsewhere on the chromosome (cf. Amor et al. 2004; Cappelletti et al. 2022).

It was hypothesized that karyotype evolution is driven by meiotic drive in many animal lineages (Pardo-Manuel de Villena and Sapienza 2001; Blackmon et al. 2019), including fishes (Yoshida and Kitano 2012; Molina et al. 2014), particularly by a nonrandom segregation of rearranged chromosome in female meiosis of heterokaryotypes, due to inherent asymmetry of female meiosis and polarity of a meiotic spindle. Stronger spindles should bind bigger centromeres (Chmátal et al. 2014; Akera et al. 2019; Kursel and Malik 2018; Kumon and Lampson 2022). Yet the direction of the nonrandom segregation is not set in stone. Reversals of spindle polarity supposedly occurred in many phylogenetic groups, which could explain differences in trends of karyotype evolution between related taxa (Pardo-Manuel de Villena and Sapienza 2001; Yoshida and Kitano 2012; Blackmon et al. 2019). It is tempting to speculate that distinct distribution of satDNAs in representatives of Southern and Coastal clade of the genus *Nothobranchius* results from meiotic drive and changes in direction of the nonrandom segregation with a stronger egg spindle pole in *N. furzeri* and *N. kadleci* than in the other species under study, as they have considerably larger pericentromeric heterochromatin blocks comprising NkadSat01-77 and NfurSat01-348 in all chromosomes but the Y chromosome. Differences between X- and Y-linked pericentromeric heterochromatin reported in *N. furzeri* and *N. kadleci* (Štundlová et al. 2022) may be explained by the absence of centromere drive on the Y chromosome as it is never transmitted via female meiosis (cf. Yoshida and Kitano 2012; Pokorná et al. 2014). Identification of repeats associated with centromeres in the Coastal clade presents an opportunity to test this hypothesis as *N. guentheri* has a multiple sex chromosome system of the X_1_X_2_Y type, in which neo-Y and one of the X chromosomes can be identified by CMA_3_ staining (Supplementary Fig. 3J). However, FISH with NrubSat01-48 was not informative as it failed to detect any satDNA clusters on both the neo-Y and the CMA_3_-positive X chromosome.

Our cytogenetic and bioinformatic data revealed a fast turnover of satDNAs associated with (peri)centromeres and distinct trends in their evolution in two clades of the *Nothobranchius* killifishes. Among teleosts, analogous example of turnover of (peri)centromeric satellites has been reported, e.g., in Neotropical genus *Triportheus*, where the repeat with a putative centromeric function was the most abundant one found in the genome of *T. auritus* (Kretschmer et al. 2022). Also in our present study, the most abundant satDNA monomer(s) in particular *Nothobranchius* species might, given their chromosomal distribution, represent candidate(s) for being functional centromeric repeats. Nevertheless, satellitome studies in other Neotropical fishes suggested that such correlation might not always hold and the situation may be more complex (de Silva et al. 2017; Serrano-Freitas et al. 2020). Further research is needed to assess the contribution of *Nothobranchius* satDNA to centromere function and to test for a role of meiotic drive in shaping molecular composition of centromeric heterochromatin. To do so, it is necessary to confirm that the candidate satDNA monomers indeed represent functional centromeric repeats. A putative CENP-B box motif was identified in NfurSat01-348 satellite. As in other fish species, its sequence showed overall 0.47 identity to the human CENP-B box (Supplementary Fig. 4), although different nucleotide positions are conserved in various fishes (Cech and Peichel 2015; Varadharajan et al. 2019; Suntronpong et al. 2020). However, the CENP-B motif was not recovered in any other killifish satellites associated with centromeres, including NrubSat01-48. In the next step, the interaction between the candidate satDNA monomers and centromeric protein CENP-A needs to be confirmed, e.g., by a chromatin immunoprecipitation-sequencing (ChIP-seq) analysis (cf. Ávila Robledillo et al. 2018; Hartley et al. 2021; Despot-Slade et al. 2022).

### Supplementary Information

Below is the link to the electronic supplementary material.

Supplementary file1 (PDF 1.54 MB)

## Data Availability

DNA sequence data was deposited in Sequence Read Archive (SRA) under the BioProject accession no. PRJNA991117. All other relevant data are within the paper and its Supporting Information file.

## Abbreviations

*2n*: Diploid chromosome number
*a*: Acrocentric chromosome
*ChIP-seq*: chromatin immunoprecipitation-sequencing
*CMA*_*3*_: Chromomycin A_3_
*DABCO*: 1,4-Diazabicyclo(2.2.2)-octane
*DAPI*: 4′,6-Diamidino-2-phenylindole
*FISH*: Fluorescence in situ hybridization
*gDNA*: Genomic DNA
*m*: Metacentric chromosome
*ND-FISH*: Non-denaturing FISH
*PBS*: Phosphate-buffered saline
*rDNA*: Ribosomal DNA
*RT*: Room temperature
*satDNA*: Satellite DNA
*SRA*: Sequence Read Archive
*SSC*: Saline-sodium citrate
*st*: Subtelocentric chromosome

## References

[CR1] Akera T, Trimm E, Lampson MA (2019). Molecular strategies of meiotic cheating by selfish centromeres. Cell.

[CR2] Amor DJ, Bentley K, Ryan J, Perry J, Wong L, Slater H, Choo KA (2004). Human centromere repositioning “in progress”. Proc Natl Acad Sci USA.

[CR3] Andrews S (2010) FastQC: a quality control tool for high throughput sequence data [Online]. http://www.bioinformatics.babraham.ac.uk/projects/fastqc/. Accessed 25 Sept 2019

[CR4] Ávila Robledillo L, Koblížková A, Novák P, Böttinger K, Vrbová I, Neumann P, Schubert I, Macas J (2018). Satellite DNA in *Vicia*
*faba* is characterized by remarkable diversity in its sequence composition, association with centromeres, and replication timing. Sci Rep.

[CR5] Ávila Robledillo L, Neumann P, Koblížková A, Novák P, Vrbová I, Macas J (2020). Extraordinary sequence diversity and promiscuity of centromeric satellites in the legume tribe Fabeae. Mol Biol Evol.

[CR6] Bartáková V, Reichard M, Blažek R, Polačik M, Bryja J (2015). Terrestrial fishes: rivers are barriers to gene flow in annual fishes from the African savanna. J Biogeogr.

[CR7] Berois N, García G, de Sá RO (2016). Annual fishes: life history strategy, diversity and evolution.

[CR8] Bertollo LAC, Cioffi MB, Moreira-Filho O (2015) Direct chromosome preparation from freshwater teleost fishes. In: Ozouf-Costaz C, Pisano E, Foresti F, and de Almeida-Toledo LF (eds) Fish cytogenetic techniques: ray-fin fishes and chondrichthyans. CRC Press, Inc, Endfield, pp 21–26. 10.1201/b18534–4

[CR9] Blackmon H, Justison J, Mayrose I, Goldberg EE (2019). Meiotic drive shapes rates of karyotype evolution in mammals. Evolution.

[CR10] Blažek R, Polačik M, Reichard M (2013). Rapid growth, early maturation and short generation time in African annual fishes. EvoDevo.

[CR11] Blažek R, Polačik M, Kačer P, Cellerino A, Řežucha R, Methling C, Tomášek O, Syslová K, Terzibasi Tozzini E, Albrecht T, Vrtílek M, Reichard M (2017). Repeated intraspecific divergence in life span and aging of African annual fishes along an aridity gradient. Evolution.

[CR12] Bracewell R, Chatla K, Nalley MJ, Bachtrog D (2019). Dynamic turnover of centromeres drives karyotype evolution in Drosophila. Elife.

[CR13] Cappelletti E, Piras FM, Sola L, Santagostino M, Abdelgadir WA, Raimondi E, Lescai F, Nergadze SG, Giulotto E (2022). Robertsonian fusion and centromere repositioning contributed to the formation of satellite-free centromeres during the evolution of zebras. Mol Biol Evol.

[CR14] Caputo V, Marchegiani F, Sorice M, Olmo E (1997). Heterochromatin heterogeneity and chromosome variability in four species of gobiid fishes (Perciformes: Gobiidae). Cytogenet Genome Res.

[CR15] Cech JN, Peichel CL (2015). Identification of the centromeric repeat in the threespine stickleback fish (*Gasterosteus*
*aculeatus*). Chromosome Res.

[CR16] Cech JN, Peichel CL (2016). Centromere inactivation on a neo-Y fusion chromosome in threespine stickleback fish. Chromosome Res.

[CR17] Cellerino A, Valenzano DR, Reichard M (2016). From the bush to the bench: the annual *Nothobranchius* fishes as a new model system in biology. Biol Rev.

[CR18] Chang SB, Yang TJ, Datema E, Van Vugt J, Vosman B, Kuipers A, Meznikova M, Szinay D, Klein Lankhorst R, Jacobsen E, de Jong H (2008). FISH mapping and molecular organization of the major repetitive sequences of tomato. Chromosome Res.

[CR19] Chmátal L, Gabriel SI, Mitsainas GP, Martínez-Vargas J, Ventura J, Searle JB, Schultz RM, Lampson MA (2014). Centromere strength provides the cell biological basis for meiotic drive and karyotype evolution in mice. Curr Biol.

[CR20] Conte MA, Joshi R, Moore EC, Nandamuri SP, Gammerdinger WJ, Roberts RB, Carleton KL, Lien S, Kocher T (2019). Chromosome-scale assemblies reveal the structural evolution of African cichlid genomes. Gigascience.

[CR21] Cuadrado Á, Jouve N (2010). Chromosomal detection of simple sequence repeats (SSRs) using nondenaturing FISH (ND-FISH). Chromosoma.

[CR22] Cui R, Medeiros T, Willemsen D, Iasi LNM, Collier GE, Graef M, Reichard M, Valenzano DR (2019). Relaxed selection limits lifespan by increasing mutation load. Cell.

[CR23] de Lima LG, Ruiz-Ruano FJ (2022). In-depth satellitome analyses of 37 *Drosophila* species illuminate repetitive DNA evolution in the *Drosophila* genus. Genome Biol Evol.

[CR24] de Silva DMZA, Utsunomia R, Ruiz-Ruano FJ, Daniel SN, Porto-Foresti F, Hashimoto DT, Oliveira C, Camacho JPM, Foresti F (2017). High-throughput analysis unveils a highly shared satellite DNA library among three species of fish genus *Astyanax*. Sci Rep.

[CR25] Despot-Slade E, Širca S, Mravinac B, Castagnone-Sereno P, Plohl M, Meštrović N (2022). Satellitome analyses in nematodes illuminate complex species history and show conserved features in satellite DNAs. BMC Biol.

[CR26] Dorn A, Musilová Z, Platzer M, Reichwald K, Cellerino A (2014). The strange case of East African annual fishes: aridification correlates with diversification for a savannah aquatic group?. BMC Evol Biol.

[CR27] Ewulonu UK, Haas R, Turner B (1985). A multiple sex chromosome system in the annual killfish, *Nothobranchius*
*guentheri*. Copeia.

[CR28] Feliciello I, Akrap I, Brajkovi J, Zlatar I, Ugarković Đ (2014). Satellite DNA as a driver of population divergence in the red flour beetle *Tribolium*
*castaneum*. Genome Biol Evol.

[CR29] Ferreira IA, Poletto AB, Kocher TD, Mota-Velasco JC, Penman DJ, Martins C (2010). Chromosome evolution in african cichlid fish: contributions from the physical mapping of repeated DNAs. Cytogenet Genome Res.

[CR30] Fricke R, Eschmeyer WN, Van der Laan R (eds) (2023) Eschmeyer’s catalog of fishes: genera, species, references. http://researcharchive.calacademy.org/research/ichthyology/catalog/fishcatmain.asp. Accessed 3 May 2023

[CR31] Furness AI (2016). The evolution of an annual life cycle in killifish: adaptation to ephemeral aquatic environments through embryonic diapause. Biol Rev Camb Philos Soc.

[CR32] Gamba R, Fachinetti D (2020). From evolution to function: two sides of the same CENP-B coin?. Exp Cell Res.

[CR33] Garrido-Ramos MA (2017). Satellite DNA: an evolving topic. Genes.

[CR34] Goes CAG, dos Santos RZ, Aguiar WRC, Alves DCV, Silva DMZA, Foresti F, Oliveira C, Utsunomia R, Porto-Foresti F (2022). Revealing the satellite DNA history in *Psalidodon* and *Astyanax* characid fish by comparative satellitomics. Front Genet.

[CR35] Goes CAG, dos Santos N, Rodrigues PHM, Stornioli JHF, da Silva AB, dos Santos RZ, Vidal JAD, Silva DMZA, Artoni RF, Foresti F (2023). The satellite DNA catalogues of two Serrasalmidae (Teleostei, Characiformes): conservation of general satDNA features over 30 million years. Genes.

[CR36] Haaf T, Schmid M (1984). An early stage of ZZ/ZW sex chromosomes differentiation in *Poecilia*
*sphenops* var. *melanistica* (Poeciliidae, Cyprinodontiformes). Chromosoma.

[CR37] Hartley G, O’Neill RJ (2019). Centromere repeats: hidden gems of the genome. Genes.

[CR38] Hartley GA, Okhovat M, Neill RJO (2021). Comparative analyses of gibbon centromeres reveal dynamic genus-specific shifts in repeat composition. Mol Biol Evol.

[CR39] Henikoff S, Ahmad K, Malik HS (2001). The centromere paradox: stable inheritance with rapidly evolving DNA. Science.

[CR40] Hu CK, Brunet A (2018). The African turquoise killifish: a research organism to study vertebrate aging and diapause. Aging Cell.

[CR41] Ichikawa K, Tomioka S, Suzuki Y, Nakamura R, Doi K, Yoshimura J, Kumagai M, Inoue Y, Uchida Y, Irie N, Takeda H, Morishita S (2017). Centromere evolution and CpG methylation during vertebrate speciation. Nat Commun.

[CR42] Kim NS, Armstrong KC, Fedak G, Ho K, Park NI (2002). A microsatellite sequence from the rice blast fungus (*Magnaporthe*
*grisea*) distinguishes between the centromeres of *Hordeum*
*vulgare* and *H*. *bulbosum* in hybrid plants. Genome.

[CR43] Kligerman AD, Bloom SE (1977). Rapid chromosome preparations from solid tissues of fishes. J Fish Res Board Can.

[CR44] Kretschmer R, Goes CAG, Bertollo LAC, Ezaz T, Porto-Foresti F, Toma GA, Utsunomia R, Cioffi MB (2022). Satellitome analysis illuminates the evolution of ZW sex chromosomes of Triportheidae fishes (Teleostei: Characiformes). Chromosoma.

[CR45] Krysanov E, Demidova T (2018). Extensive karyotype variability of African fish genus *Nothobranchius* (Cyprinodontiformes). Comp Cytogenet.

[CR46] Krysanov E, Demidova T, Nagy B (2016). Divergent karyotypes of the annual killifish genus *Nothobranchius* (Cyprinodontiformes, Nothobranchiidae). Comp Cytogenet.

[CR47] Krysanov EY, Nagy B, Watters BR, Sember A, Simanovsky SA (2023). Karyotype differentiation in the *Nothobranchius*
*ugandensis* species group (Teleostei, Cyprinodontiformes), seasonal fishes from the east African inland plateau, in the context of phylogeny and biogeography. Comp Cytogenet.

[CR48] Kumon T, Lampson MA (2022). Evolution of eukaryotic centromeres by drive and suppression of selfish genetic elements. Semin Cell Dev Biol.

[CR49] Kursel LE, Malik HS (2018). The cellular mechanisms and consequences of centromere drive. Curr Opin Cell Biol.

[CR50] Levan AK, Fredga K, Sandberg AA (1964). Nomenclature for centromeric position on chromosomes. Hereditas.

[CR51] Lower SS, McGurk MP, Clark AG, Barbash DA (2018). Satellite DNA evolution: old ideas, new approaches. Curr Opin Genet Dev.

[CR52] Lukšíková K, Pavlica T, Altmanová M, Štundlová J, Pelikánová Š, Simanovsky SA, Yu KE, Jankásek M, Hiřman M, Reichard M, Ráb P, Sember A (2023) Conserved satellite DNA motif and lack of interstitial telomeric sites in highly rearranged African *Nothobranchius* killifish karyotypes. J Fish Biol 1–14. 10.1111/jfb.15550 10.1111/jfb.15550 37661806

[CR53] Martin M (2011). Cutadapt removes adapter sequences from high-throughput sequencing reads. EMBnet J.

[CR54] Masumoto H, Masukata H, Muro Y, Nozaki N, Okazaki T (1989). A human centromere antigen (CENP-B) interacts with a short specific sequence in alphoid DNA, a human centromeric satellite. J Cell Biol.

[CR55] Mayr B, Ráb P, Kalat M (1985). Localisation of NORs and counterstain-enhanced fluorescence studies in *Perca*
*fluviatilis* (Pisces, Percidae). Genetica.

[CR56] McKinley KL, Cheeseman IM (2016). The molecular basis for centromere identity and function. Nat Rev Mol Cell Biol.

[CR57] Melters DP, Bradnam KR, Young HA, Young HA, Telis N, May MR, Ruby JG, Sebra R, Peluso P, Eid J, Rank D, Garcia JF (2013). Comparative analysis of tandem repeats from hundreds of species reveals unique insights into centromere evolution. Genome Biol.

[CR58] Molina WF, Martinez PA, Bertollo LAC, Bidau CJ (2014). Evidence for meiotic drive as an explanation for karyotype changes in fishes. Mar Genomics.

[CR59] Mora P, Pita S, Montiel EE, Rico-Porras JM, Palomeque T, Panzera F, Lorite P (2023). Making the genome huge: the case of *Triatoma*
*delpontei*, a Triatominae species with more than 50% of its genome full of satellite DNA. Genes.

[CR60] Nagy B, Watters BR (2021). A review of the conservation status of seasonal *Nothobranchius* fishes (Teleostei: Cyprinodontiformes), a genus with a high level of threat, inhabiting ephemeral wetland habitats in Africa. Aquat Conserv.

[CR61] Nishihara H, Stanyon R, Tanabe H, Koga A (2021). Replacement of owl monkey centromere satellite by a newly evolved variant was a recent and rapid process. Genes Cells.

[CR62] Novák P, Neumann P, Macas J (2020). Global analysis of repetitive DNA from unassembled sequence reads using RepeatExplorer2. Nat Protoc.

[CR63] Palacios-Gimenez OM, Milani D, Song H, Marti DA, López-León MD, Ruiz-Ruano FJ, Camacho JPM, Cabral-de-Mello DC (2020). Eight million years of satellite DNA evolution in grasshoppers of the genus *Schistocerca* illuminate the ins and outs of the library hypothesis. Genome Biol Evol.

[CR64] Pardo-Manuel De Villena F, Sapienza C (2001). Nonrandom segregation during meiosis: the unfairness of females. Mamm Genome.

[CR65] Peona V, Kutschera VE, Blom MP, Irestedt M, Suh A (2022). Satellite DNA evolution in Corvoidea inferred from short and long reads. Mol Ecol.

[CR66] Pita S, Panzera F, Mora P, Vela J, Cuadrado Á, Sánchez A, Palomeque T, Lorite P (2017). Comparative repeatome analysis on *Triatoma*
*infestans* Andean and non-Andean lineages, main vector of Chagas disease. PLoS One.

[CR67] Plohl M, Meštrović N, Mravinac B (2012). Satellite DNA evolution. Genome Dyn.

[CR68] Pokorná M, Altmanová M, Kratochvíl L (2014). Multiple sex chromosomes in the light of female meiotic drive in amniote vertebrates. Chromosome Res.

[CR69] Ráb P, Roth P, Balicek P, Forejt J, Rubeš J (1988). Cold-blooded vertebrates. Methods of chromosome analysis.

[CR70] Reichard M, Giannetti K, Ferreira T, Maouche A, Vrtílek M, Polačik M, Blažek R, Ferreira MG (2022). Lifespan and telomere length variation across populations of wild-derived African killifish. Mol Ecol.

[CR71] Reichwald K, Lauber C, Nanda I, Kirschner J, Hartmann N, Schories S, Gausmann U, Taudien S, Schilhabel MB, Szafranski K, Glöckner G, Schmid M (2009). High tandem repeat content in the genome of the short-lived annual fish *Nothobranchius*
*furzeri*: a new vertebrate model for aging research. Genome Biol.

[CR72] Reichwald K, Petzold A, Koch P, Downie BR, Hartmann N, Pietsch S, Baumgart M, Chalopin D, Felder M, Bens M, Sahm A, Szafranski K (2015). Insights into sex chromosome evolution and aging from the genome of a short-lived fish. Cell.

[CR73] Ruiz-Ruano FJ, López-León MD, Cabrero J, Camacho JPM (2016). High-throughput analysis of the satellitome illuminates satellite DNA evolution. Sci Rep.

[CR74] Šatović-Vukšić E, Plohl M (2023). Satellite DNAs—from localized to highly dispersed genome components. Genes.

[CR75] Sember A, Bohlen J, Šlechtová V, Altmanová M, Symonová R, Ráb P (2015). Karyotype differentiation in 19 species of river loach fishes (Nemacheilidae, Teleostei): extensive variability associated with rDNA and heterochromatin distribution and its phylogenetic and ecological interpretation. BMC Evol Biol.

[CR76] Serrano-Freitas ÉA, Silva DMZA, Ruiz-Ruano FJ, Utsunomia R, Araya-Jaime C, Oliveira C, Camacho JPM, Foresti F (2020). Satellite DNA content of B chromosomes in the characid fish *Characidium*
*gomesi* supports their origin from sex chromosomes. Mol Genet Genomics.

[CR77] Sola L, Rossi AR, Iaselli V, Rasch EM, Monaco PJ (1992). Cytogenetics of bisexual/unisexual species of Poecilia. II. Analysis of heterochromatin and nucleolar organizer regions in Poecilia mexicana mexicana by C-banding and DAPI, quinacrine, chromomycin A3, and silver staining. Cytogenet Cell Genet.

[CR78] Stornioli JHF, Goes CAG, Calegari RM, dos Santos RZ, Giglio LM, Foresti F, Oliveira C, Penitente M, Porto-Foresti F, Utsunomia R (2021). The B chromosomes of *Prochilodus*
*lineatus* (Teleostei, Characiformes) are highly enriched in satellite DNAs. Cells.

[CR79] Štundlová J, Hospodářská M, Lukšíková K, Voleníková A, Pavlica T, Altmanová M, Richter A, Reichard M, Dalíková M, Pelikánová Š, Marta A, Simanovsky SA (2022). Sex chromosome differentiation via changes in the Y chromosome repeat landscape in African annual killifishes *Nothobranchius*
*furzeri* and *N. kadleci*. Chromosome Res.

[CR80] Suntronpong A, Kugou K, Masumoto H, Srikulnath K, Ohshima K, Hirai H, Koga A (2016). CENP-B box, a nucleotide motif involved in centromere formation, occurs in a New World monkey. Biol Lett.

[CR81] Suntronpong A, Singchat W, Kruasuwana W, Prakhongcheep O, Sillapaprayoon S, Muangmai N, Somyong S, Indananda C, Kraichak E, Peyachoknagul S, Srikulnath K (2020). Characterization of centromeric satellite DNAs (MALREP) in the Asian swamp eel (*Monopterus*
*albus*) suggests the possible origin of repeats from transposable elements. Genomics.

[CR82] Talbert PB, Henikoff S (2020). What makes a centromere?. Exp Cell Res.

[CR83] Tao W, Xu L, Zhao L, Zhu Z, Wu X, Min Q, Wang D, Zhou Q (2021). High-quality chromosome-level genomes of two tilapia species reveal their evolution of repeat sequences and sex chromosomes. Mol Ecol Resour.

[CR84] Terzibasi Tozzini E, Cellerino A (2020). Nothobranchius annual killifishes. Evodevo.

[CR85] Thakur J, Packiaraj J, Henikoff S (2021). Sequence, chromatin and evolution of satellite DNA. Int J Mol Sci.

[CR86] The Galaxy Community (2022). The Galaxy platform for accessible, reproducible and collaborative biomedical analyses: 2022 update. Nucleic Acids Res.

[CR87] van der Merwe PDW, Cotterill FPD, Kandziora M, Watters BR, Nagy B, Genade T, Flügel TJ, Svendsen DS, Bellstedt DU (2021). Genomic fingerprints of palaeogeographic history: the tempo and mode of rift tectonics across tropical Africa has shaped the diversification of the killifish genus *Nothobranchius* (Teleostei: Cyprinodontiformes). Mol Phylogenet Evol.

[CR88] Varadharajan S, Rastas P, Lӧytynoja A, Matschiner M, Calboli FCF, Guo B, Nederbragt AJ, Jakobsen KS, Merilä J (2019). A high-quality assembly of the nine-spined stickleback (*Pungitius*
*pungitius*) genome. Genome Biol Evol.

[CR89] Völker M, Ráb P, Ozouf-Costaz C, Pisano E, Foresti F, de Almeida-Toledo LF (2015). Direct chromosome preparation from regenerating fin tissue. Fish cytogenetic techniques: ray-fin fishes and chondrichthyans.

[CR90] Völker M, Ráb P, Kullmann H (2008). Karyotype differentiation in *Chromaphyosemion* killifishes (Cyprinodontiformes, Nothobranchiidae): patterns, mechanisms, and evolutionary implications. Biol J Linn Soc.

[CR91] Vondrak T, Ávila Robledillo L, Novák P, Koblížková A, Neumann P, Macas J (2020). Characterization of repeat arrays in ultra-long nanopore reads reveals frequent origin of satellite DNA from retrotransposon-derived tandem repeats. Plant J.

[CR92] Watters BR, Cooper BJ, Wildekamp RH (2008). Description of Nothobranchius cardinalis spec. nov. (Cyprinodontiformes: Aplocheilidae), an annual fish from the Mbwemkuru River basin, Tanzania. J Am Killifsh Ass.

[CR93] Watters BR, Nagy B, van der Merwe PDW, Cotterill FPD, Bellstedt DU (2020). Redescription of the seasonal killifish species *Nothobranchius*
*ocellatus* and description of a related new species *Nothobranchius*
*matanduensis*, from eastern Tanzania (Teleostei: Nothobranchiidae). Ichthyol Explor Freshw.

[CR94] Wildekamp RH (1996). A world of killies. Atlas of the oviparous cyprinodontiform fishes of the world (Vol. III).

[CR95] Wildekamp RH (2004) A world of killies – atlas of the oviparous cyprinodontiform fishes of the world (Vol. 4). The American Killifish Association, Elyria, Ohio

[CR96] Willemsen D, Cui R, Reichard M, Valenzano DR (2020). Intra-species differences in population size shape life history and genome evolution. Elife.

[CR97] Yano CF, Bertollo LAC, Ezaz T, Trifonov V, Sember A, Liehr T, Cioffi MB (2017). Highly conserved Z and molecularly diverged W chromosomes in the fish genus *Triportheus* (Characiformes, Triportheidae). Heredity.

[CR98] Yoshida K, Kitano J (2012). The contribution of female meiotic drive to the evolution of neo-sex chromosomes. Evolution.

[CR99] Ziemniczak K, Barros AV, Rosa KO, Nogaroto V, Almeida MC, Cestari MM, Moreira-Filho O, Artoni RF, Vicari MR (2012). Comparative cytogenetics of Loricariidae (Actinopterygii: Siluriformes): emphasis in Neoplecostominae and Hypoptopomatinae. Ital J Zool.

