# Supplementary material for: Fast satellite DNA evolution in Nothobranchius annual killifishes
Source: Chromosome Res. 2023 Nov 21;31(4):33. doi: 10.1007/s10577-023-09742-8 (PMC10661780; doi:10.1007/s10577-023-09742-8)
Supplement: Supplementary file 1 — Supplementary file1 (PDF 1.54 MB) [file 10577_2023_9742_MOESM1_ESM.pdf]

Supporting information for

Fast satellite DNA evolution in *Nothobranchius* annual killifishes

Journal: Chromosome Research

Anna Voleníková, Karolína Lukšíková, Pablo Mora, Tomáš Pavlica, Marie Altmanová, Jana Štundlová, Šárka Pelikánová, Sergey A. Simanovsky, Marek Jankásek, Martin Reichard, Petr Nguyen*, Alexandr Sember*

* Correspondence:

sember@iapg.cas.cz; Institute of Animal Physiology and Genetics, Czech Academy of Sciences, Liběchov, Czech Republic

petr.nguyen@prf.jcu.cz; University of South Bohemia, Faculty of Science, České Budějovice, Czech Republic

The PDF file includes:

Supplementary Fig. 1
Supplementary Fig. 2
Supplementary Fig. 3
Supplementary Fig. 4
Supplementary Fig. 5
Supplementary Fig. 6
Supplementary Fig. 7
Supplementary Fig. 8
Supplementary Table. 1

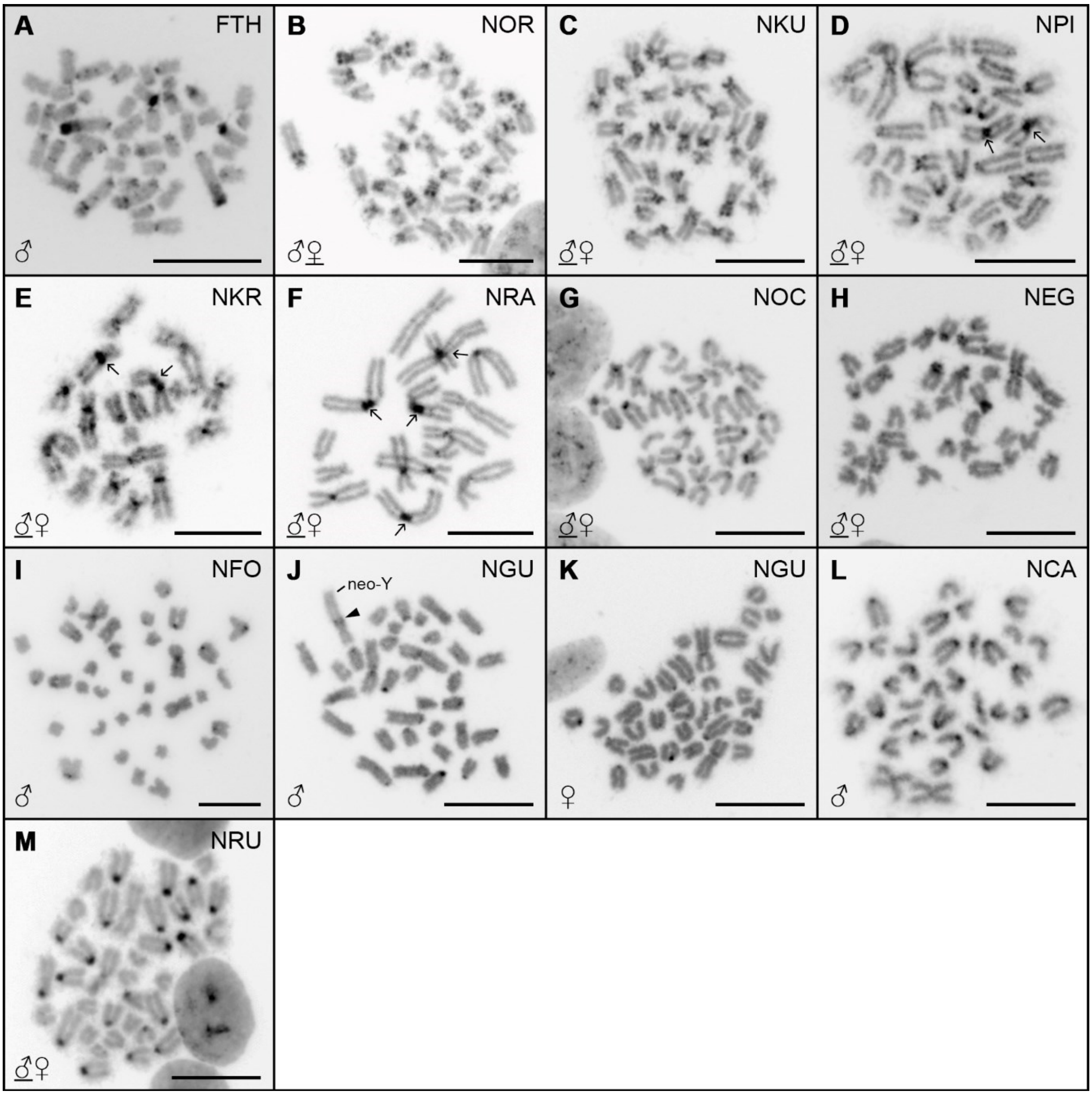

Supplementary Fig. 1 Mitotic metaphases of *F. thierryi* and *Nothobranchius* spp. after C-banding. Sex of the studied individuals is indicated and eventually underlined where both sexes (if studied) presented the same distribution pattern (i.e. except for *N. guentheri*; J, K). Arrows indicate examples of huge (peri)centromeric heterochromatin blocks in expected fusion sites on large metacentric chromosomes in the Southern-clade species (D–F). Neo-Y chromosome in *N. guentheri* male (J) is identified based on its distinctive morphology; full arrowhead points to the heterochromatin block representing an assumed fusion site. Chromosomes stained with DAPI (inverted colors). Species order reflects their phylogenetic relationships (for details, see Fig. 2 in the main text). Species acronyms are summarized in Table 1 in the main text. Scale bar = 10 μm

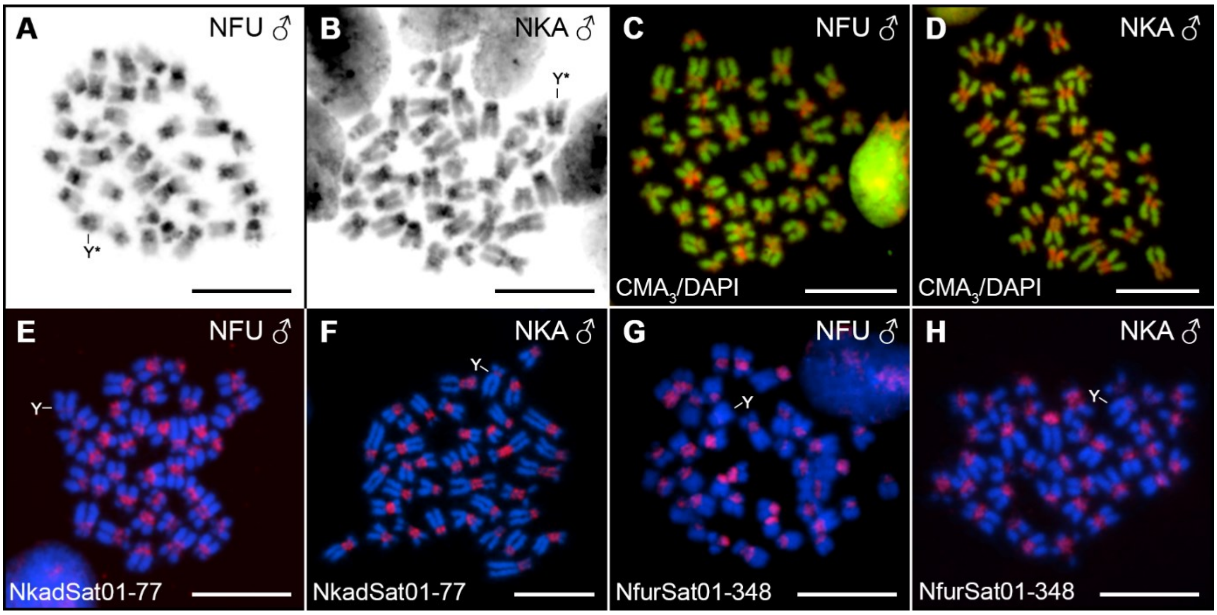

Supplementary Fig. 2 Mitotic metaphases of *N. furzeri* and *N. kadleci* after various cytogenetic treatments. (A, B) C-banding, (C, D), CMA_3/DAPI staining, (E, F) FISH with NkadSat01-77 probe, (G, H), FISH with NfurSat01-348 probe. All results originate from the experiments published in our previous work (Štundlová et al. 2022) and serve here as a direct comparison of patterns revealed in the present study. The presented metaphases belong to male representatives of the same populations as used in the present study: *N. furzeri* MZCS-222 and *N. kadleci* MZCS-91. Assumed Y sex chromosomes are marked if detectable. Chromosomes were counterstained with DAPI (blue). Images follow the same color coding as the remaining supplementary figures in the present study (depending on the method used). Species acronyms are summarized in Table 1 in the main text. Scale bar = 10 μm

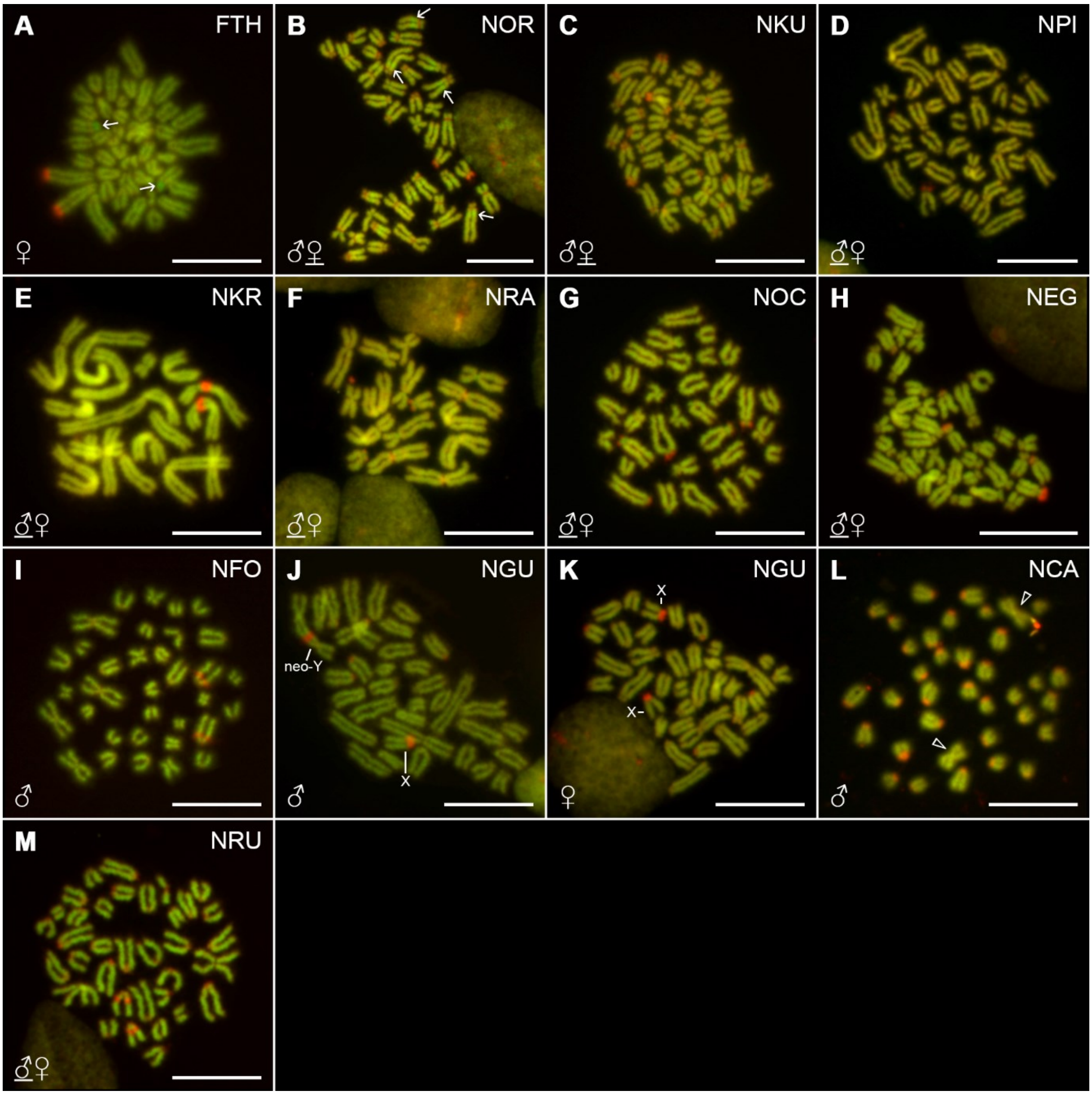

Supplementary Fig. 3 Mitotic metaphases of *F. thierryi* and *Nothobranchius* spp. after CMA_3/DAPI staining. Sex of the studied individuals is indicated and eventually underlined where both sexes (if studied) presented the same distribution pattern (i.e. except for *N. guentheri*; J, K). For better contrast, images were pseudocolored in red (for CMA_3) and green (for DAPI). Neo-Y chromosome and one of the X chromosomes in *N. guentheri* male (J) are identified based on distinctive morphology and shared strong CMA_3^+ signals, respectively. Both X homologs are marked in *N. guentheri* female (K). In *N. cardinalis* (L) empty arrowheads point to the only metacentric chromosomes in the complement which are also the only elements lacking the (peri)centromeric CMA_3 signals. Arrows point to examples of rarely observed pronounced AT-rich regions (A, B). Species order reflects their phylogenetic relationships (for details, see Fig. 2 in the main text). Species acronyms are summarized in Table 1 in the main text. Scale bar = 10 μm

		*	*	*	*					*			*	*	*	*		**identity**
H. sapiens	**C**	**T**	**T**	**C**	**G**	**T**	**T**	**G**	**G**	**A**	**A**	**A**	**C**	**G**	**G**	**G**	**A**	
G. aculeatus	A	A	A	G	G	T	T	G	G	A	A	A	A	C	T	A	T	0.47
P. pungitus	T	A	G	T	T	T	T	A	G	A	A	C	C	T	G	A	N	0.44
M. albus	A	T	A	A	C	A	C	T	G	A	A	A	T	G	G	T	T	0.41
N. furzeri	C	T	T	C	G	T	G	C	A	A	C	A	T	A	A	T	T	0.47

Supplementary Fig. 4 A putative CENP-B box motif in Nfu-SatB satellite in the turquoise killifish, *Nothobranchius furzeri*. Alignment shows 0.47 identity to the human CENP-B box sequence, which is comparable to those characterized in threespine stickleback (*Gasterosteus aculeatus*; 0.47 identity), ninespine stickleback (*Pungitus pungitus;* 0.44 identity) and Asian swamp eel (*Monopterus albus;* 0.41 identity). Conserved nucleotides of the human CENP-B box motif are indicated in red and functionally important domains for primates (Suntronpong et al. 2016; see References in the main MS body) are marked by asterisks

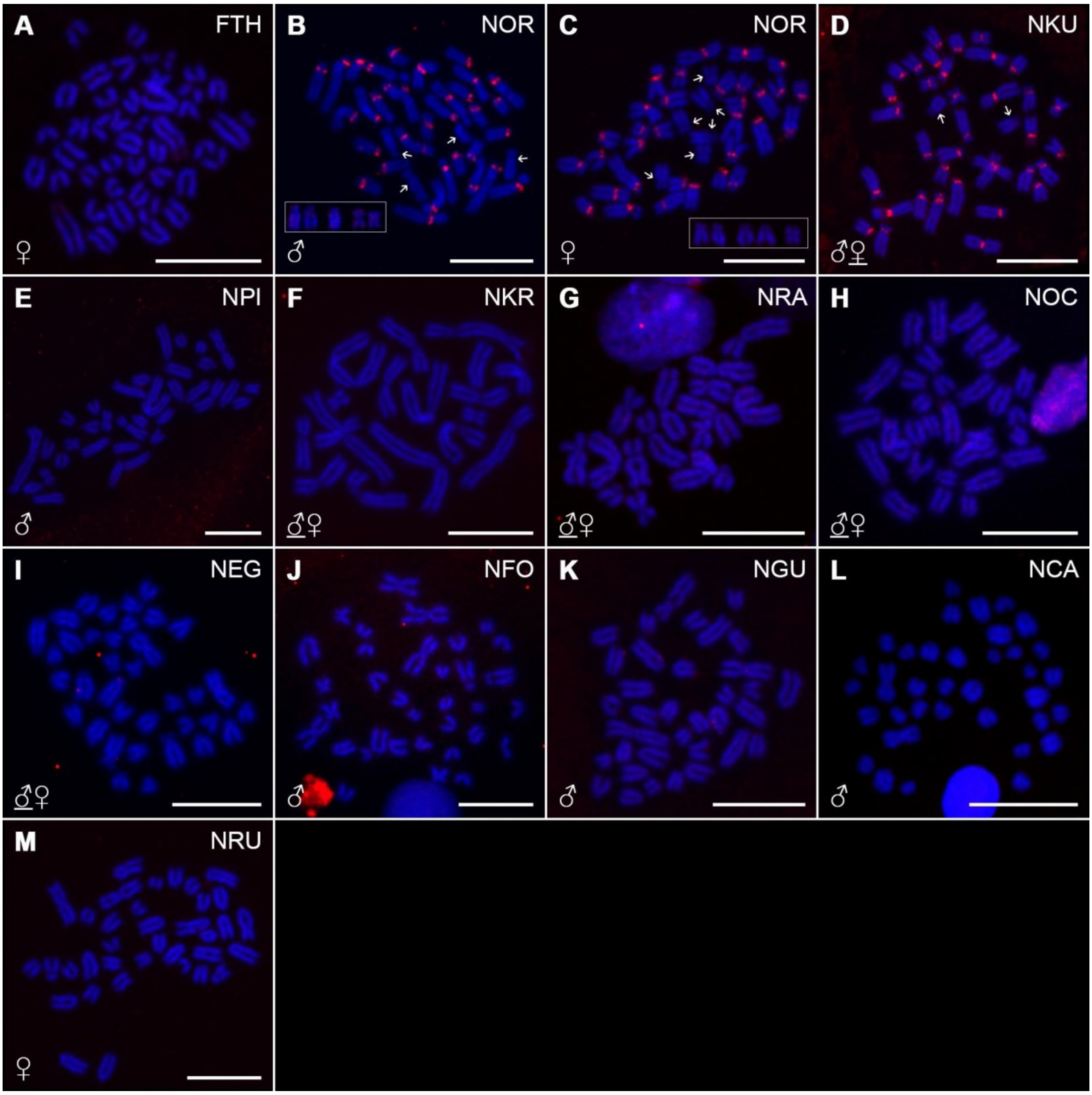

Supplementary Fig. 5 Mitotic metaphases of *F. thierryi* and *Nothobranchius* spp. after FISH with NkadSat01-77 repeat (red signals). Sex of the studied individuals is indicated and eventually underlined where both sexes (if studied) presented the same distribution pattern (i.e. except for *N. orthonotus*; B, C). In *N. orthonotus* (B, C) and *N. kuhntae* (D), arrows point to chromosomes lacking the (peri)centromeric signals. Polymorphic patterns regarding this feature are framed. Chromosomes were counterstained with DAPI (blue). Species order reflects their phylogenetic relationships (for details, see Fig. 2 in the main text). Species acronyms are summarized in Table 1 in the main text. Scale bar = 10 µm

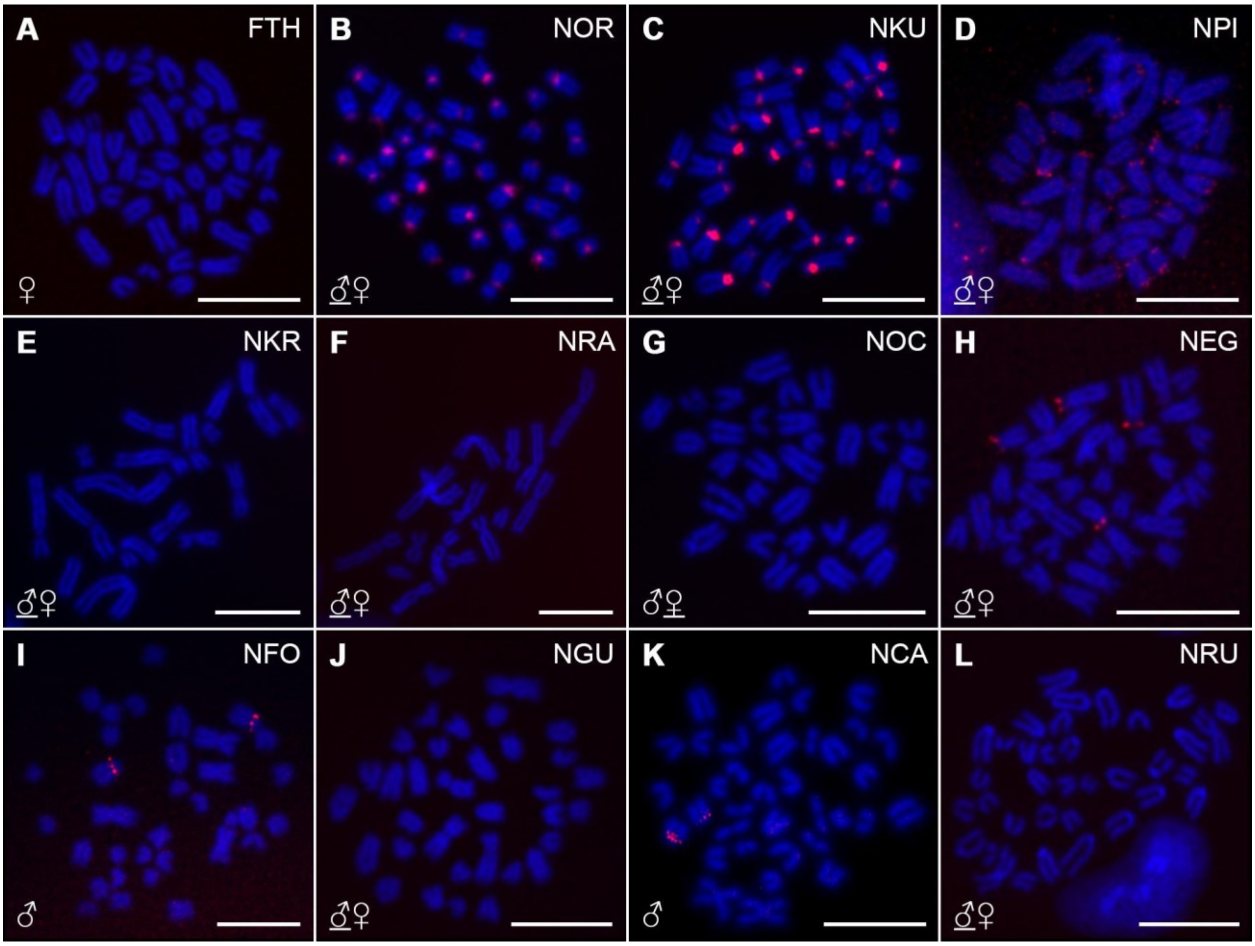

Supplementary Fig. 6 Mitotic metaphases of *F. thierryi* and *Nothobranchius* spp. after FISH with NfurSat01-348 repeat (red signals). Sex of the studied individuals is indicated and eventually underlined where both sexes (if studied) presented the same distribution pattern. Chromosomes were counterstained with DAPI (blue). Species order reflects their phylogenetic relationships (for details, see Fig. 2 in the main text). Species acronyms are summarized in Table 1 in the main text. Scale bar = 10 μm

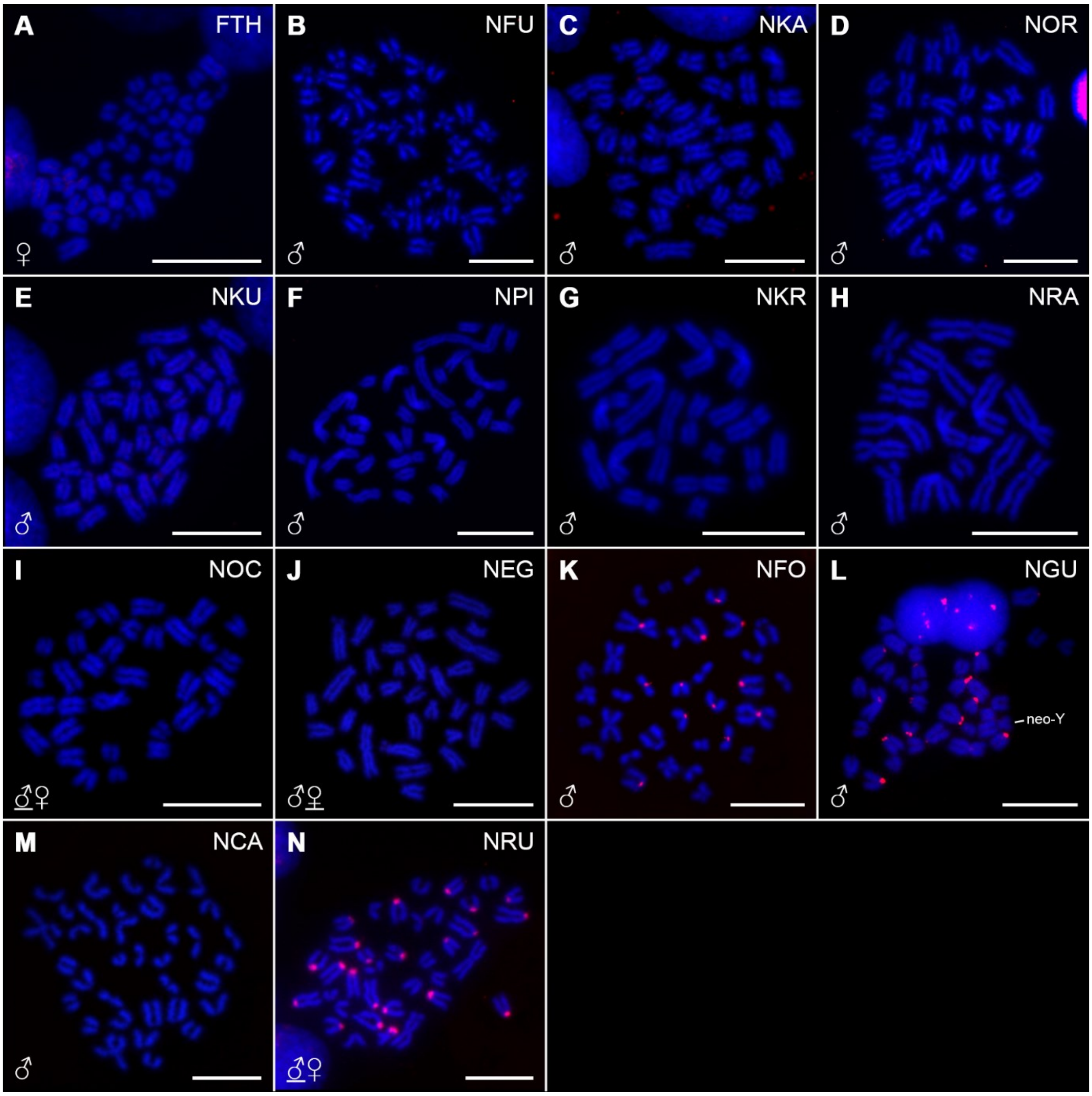

Supplementary Fig. 7 Mitotic metaphases of *F. thierryi* and *Nothobranchius* spp. after FISH with NrubSat01-48 repeat (red signals). Sex of the studied individuals is indicated and eventually underlined where both sexes (if studied) presented the same distribution pattern. Neo-Y chromosome in *N. guentheri* male (L) is identified based on distinctive morphology. Chromosomes were counterstained with DAPI (blue). Species order reflects their phylogenetic relationships (for details, see Fig. 2 in the main text). Species acronyms are summarized in Table 1 in the main text. Scale bar = 10 μm

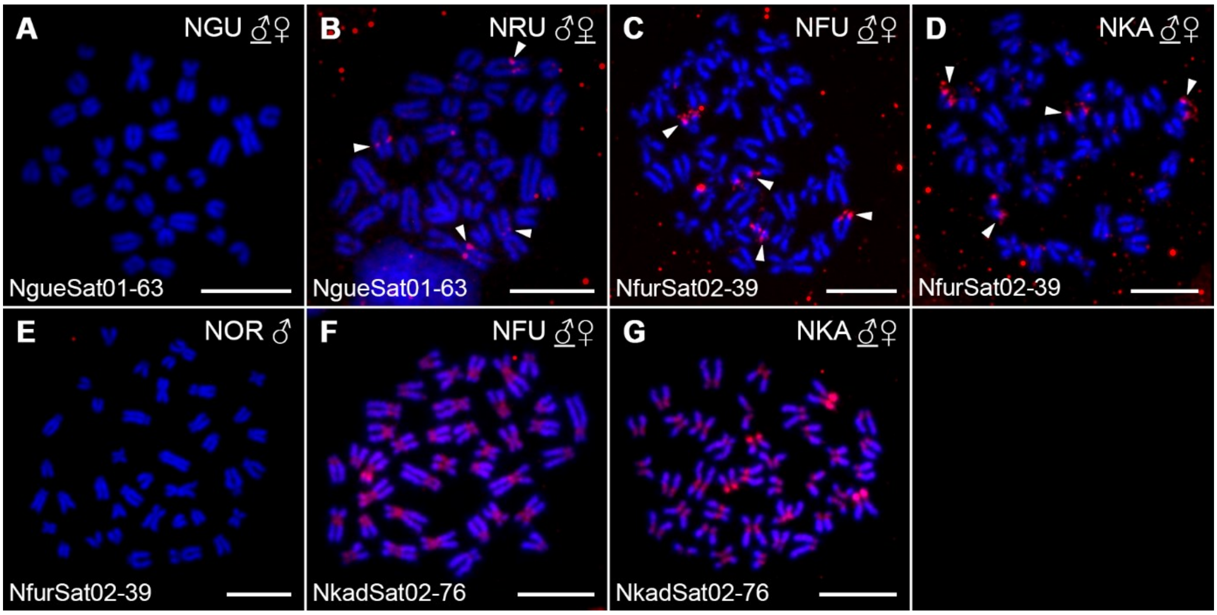

Supplementary Fig. 8 Mitotic metaphases of selected *Nothobranchius* spp. after FISH with three different satDNA probes (red signals and arrowheads in B–D). Sex of the studied individuals is indicated and eventually underlined where both sexes (if studied) presented the same distribution pattern. Chromosomes were counterstained with DAPI (blue). Species acronyms are summarized in Table 1 in the main text. Scale bar = 10 μm

Supplementary Table 1 A detailed list of studied *Nothobranchius* killifish species with their sample sizes (N) used for each method, population/collection codes, source/geographic origin and GPS coordinates of sampling localities

Clade	Species	Code	N									Population (collection code)	Source / locality	GPS coordinates
			C-banding	CMA_3	Nkad Sat01-77	Nfur Sat01-348	Nfur Sat02-39	Nkad Sat02-76	Nrub Sat01-48	Ngue Sat01-63	Summary			
outgroup	*Fundulosoma thierryi* Ahl, 1924	FTH	1♂, 2♀	2♀	2♀	2♀	–	–	3♀	–	1♂, 3♀	aquarium strain	–	–
Southern	*Nothobranchius furzeri* Jubb, 1971	NFU	*	*	*	*	1♂, 1♀	1♂, 1♀	2♂	–	2♂, 1♀	MZCS-222	Chefu, Mozambique	21°52'24.8"S 32°48'2.3"E
	N. kadleci Reichard, 2010	NKA	*	*	*	*	1♂, 1♀	1♂, 1♀	1♂	–	1♂, 1♀	MZCS-91	Gorongosa, Mozambique	20°41'16.6"S 34°6'21.9"E
	N. orthonotus (Peters, 1844)	NOR	2♂, 3♀	1♂, 1♀	3♂, 3♀	1♂, 1♀	1♂, 2♀	–	1♂	–	3♂, 3♀	MZCS-02	Limpopo, Mozambique	24°03'48.5"S 32°43'55.9"E
	N. kuhntae (Ahl, 1926)	NKU	4♂, 1♀	1♂, 2♀	3♂, 3♀	1♂, 1♀	–	–	2♂	–	4♂, 3♀	MZCS-528	Pungwe, Mozambique	19°41'50.5"S 34°46'58.6"E
	N. pienaari Shidlovskyi, Watters & Wildekamp, 2010	NPI	2♂, 2♀	2♂, 3♀	2♂	2♂, 2♀	–	–	1♂	–	2♂, 3♀	MZCS-505	Limpopo, Mozambique	23°31'47.2"S 32°34'40.6"E
	N. krysanovi Shidlovskyi, Watters & Wildekamp, 2010	NKR	2♂, 2♀	1♂, 2♀	1♂, 2♀	1♂, 2♀	–	–	1♂	–	2♂, 2♀	aquarium strain; MZCS-249	Quelimane, Mozambique	17°48'52.2"S 36°54'49.4"E
	N. rachovii Ahl, 1926	NRA	2♂, 2♀	1♂, 1♀	1♂, 1♀	1♂, 1♀	–	–	1♂	–	2♂, 2♀	MZCS-096	Beira Airport, Mozambique	19°48'48.8"S 34°54'17.6"E
Ocellatus	*N. ocellatus* (Seegers, 1985)	NOC	1♂, 1♀	1♂, 1♀	1♂, 1♀	1♂, 1♀	–	–	1♂, 1♀	–	1♂, 1♀	–	Nyamwage, Tanzania	–
Coastal	*N. eggersi* Seegers, 1982	NEG	2♂, 1♀	1♂, 1♀	2♂, 1♀	1♂, 1♀	–	–	2♂, 1♀	–	2♂, 1♀	T52	Bagamoyo, Tanzania	6°28'55.9"S 38°54'51.5"E
	N. foerschi Wildekamp & Berkenkamp, 1979	NFO	2♂	2♂	2♂	2♂	–	–	2♂	–	2♂	CI 57	Soga, Tanzania	6°50'13.2"S 38°50'45.6"E
	N. guentheri (Pfeffer, 1983)	NGU	3♂, 3♀	4♂, 2♀	1♂, 2♀	1♂, 2♀	–	–	2♂	2♂, 1♀	4♂, 3♀	aquarium strain	Zanzibar, Tanzania	–
	N. cardinalis Watters, Cooper & Wildekamp, 2008	NCA	1♂	1♂	1♂	1♂	–	–	1♂	–	1♂	TTKSN 17-12	Matandu, Tanzania	9°30'04.0"S 38°13'49.0"E
	N. rubripinnis Seegers, 1986	NRU	1♂, 1♀	1♂, 2♀	2♀	2♂	–	–	2♂, 2♀	1♂, 2♀	2♂, 2♀	T33	Kitonga, Tanzania	7°12'40.5"S 39°10'30.9"E

* reported in Štundlová et al. 2022 (see References in the main MS body)

Species order reflects their phylogenetic relationships (for details, see Fig. 2 in the main text)
